# Analysis of a set of Australian northern brown bandicoot expressed sequence tags with comparison to the genome sequence of the South American grey short tailed opossum

**DOI:** 10.1186/1471-2164-8-50

**Published:** 2007-02-13

**Authors:** Michelle L Baker, Sandra Indiviglio, April M Nyberg, George H Rosenberg, Kerstin Lindblad-Toh, Robert D Miller, Anthony T Papenfuss

**Affiliations:** 1 Center for Evolutionary and Theoretical Immunology, Department of Biology, University of New Mexico, Albuquerque, NM 87131, USA; 2 Broad Institute of MIT and Harvard, 7 Cambridge Center, Cambridge, MA 02142, USA; 3 Bioinformatics Division, The Walter and Eliza Hall Institute of Medical Research, Parkville, Australia

## Abstract

**Background:**

Expressed sequence tags (ESTs) have been used for rapid gene discovery in a variety of organisms and provide a valuable resource for whole genome annotation. Although the genome of one marsupial, the opossum *Monodelphis domestica*, has now been sequenced, no EST datasets have been reported from any marsupial species. In this study we describe an EST dataset from the bandicoot, *Isoodon macrourus*, providing information on the transcriptional profile of the bandicoot thymus and the opportunity for a genome wide comparison between the bandicoot and opossum, two distantly related marsupial species.

**Results:**

A set of 1319 ESTs was generated from sequencing randomly chosen clones from a bandicoot thymus cDNA library. The nucleic acid and deduced amino acid sequences were compared with sequences both in GenBank and the recently completed whole genome sequence of *M. domestica*. This study provides information on the transcriptional profile of the bandicoot thymus with the identification of genes involved in a broad range of activities including protein metabolism (24%), transcription and/or nucleic acid metabolism (10%), metabolism/energy pathways (9%), immunity (5%), signal transduction (5%), cell growth and maintenance (3%), transport (3%), cell cycle (0.7%) and apoptosis (0.5%) and a proportion of genes whose function is unknown (5.8%). Thirty four percent of the bandicoot ESTs found no match with annotated sequences in any of the public databases. Clustering and assembly of the 1319 bandicoot ESTs resulted in a set of 949 unique sequences of which 375 were unannotated ESTs. Of these, seventy one unannotated ESTs aligned to non-coding regions in the opossum, human, or both genomes, and were identified as strong non-coding RNA candidates. Eighty-four percent of the 949 assembled ESTs aligned with the *M. domestica *genome sequence indicating a high level of conservation between these two distantly related marsupials.

**Conclusion:**

This study is among the first reported marsupial EST datasets with a significant inter-species genome comparison between marsupials, providing a valuable resource for transcriptional analyses in marsupials and for future annotation of marsupial whole genome sequences.

## Background

Marsupials and eutherian mammals are each other's closest relatives, having diverged from a common ancestor around 172 million years ago (MYA) [1-3]. Marsupials are most clearly distinguished from eutherians by their mode of reproduction, giving birth to young at a relatively immature stage of development and placing greater emphasis on lactation than on intrauterine development during pregnancy [4]. Much of the development that occurs *in utero *in eutherians is postnatal in marsupials, including the development of the immune system [5-9]. However, young marsupials are most vulnerable to dying not at birth, but at the time of emergence from the nest or pouch, suggesting that maternal antibodies and anti-microbials provide protection during the lactation period [4].

Although many marsupial genes have been identified using homology based approaches such as PCR and DNA-DNA hybridization, the low conservation of some genes makes them difficult to amplify and clone based solely on sequence similarity with homologs from other species [10-13]. Expressed sequence tags (ESTs) are single-pass, partial sequences of cDNA clones and have been used extensively for gene discovery and genome mapping in humans and other organisms [14-17]. ESTs also provide useful information for comparative genome analysis and yield information on gene expression profiles of specific organs and cells [15-19]. A whole genome sequence is available from one marsupial, the South American opossum (*Monodelphis domestica*). However, gene identification and mapping within the opossum genome is hampered by the comparatively limited information available on the transcriptome of any marsupial species.

The bandicoot is a member of the Peramelidae family of Australian marsupials found throughout mainland Australia, Tasmania and New Guinea. Although no genome project exists for any Peramelid, bandicoots have a number of unique characteristics which make them important model organisms for examining mammalian evolution, genetics, developmental biology and the evolution of viviparity and fetal-maternal tolerance. The relationship of bandicoots to other marsupials has been somewhat enigmatic, however, bandicoots are generally considered to be a very ancient group, separate from the rest of the Australasian marsupials and having closest affinity to the South American Caenolestid family [20]. All marsupials develop a simple yolk sac placenta during pregnancy. In addition to the yolk sac placenta, bandicoots are unique among marsupials in that they are the only group that develops a highly invasive chorio-allantoic placenta, similar to that of eutherian mammals, during the final 2 days of their 12.5 day gestation period. The post-natal growth of bandicoots is the most rapid of any marsupial, suggesting that the chorio-allantoic placenta is likely a more efficient organ of exchange than the yolk sac placenta [21]. Another unique characteristic of bandicoots is the process of chromosome inactivation. In most somatic tissues during development the paternally derived X chromosome in females and the Y chromosome in males is eliminated [22].

In an effort to identify immune related genes in the bandicoot, we carried out an analysis of ESTs using a thymus cDNA library from the northern brown bandicoot, *I. macrourus*. Due to its role in central tolerance, the thymus expresses a large percentage of the proteome and therefore allows for the identification of a broad range of genes including those involved in the immune response [23]. This study represents one of the first analyses of ESTs reported from any marsupial. In addition to providing important information on the gene profile of the bandicoot thymus, comparison of the bandicoot ESTs with the opossum genome is among the largest genome wide comparison between two marsupials reported to date. This information provides valuable resources for future studies of gene expression in bandicoots and other marsupials and will assist in the annotation of marsupial whole genome sequences.

## Results and discussion

### Overview of expressed sequence tags

**Figure 1:**
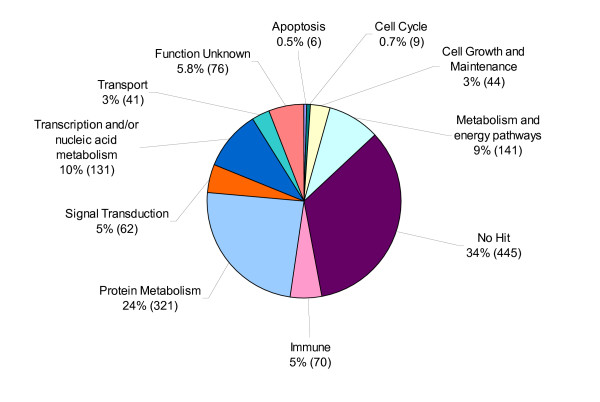
The distribution of 1319 expressed sequence tags (ESTs) from a bandicoot thymus cDNA library.

The 5' ends of 2000 randomly selected cDNA clones from the bandicoot thymus cDNA library were sequenced, yielding a total of 1319 ESTs. The average insert size of the bandicoot cDNA clones was 1.4 Kb and the average length of useable sequence obtained was 412 bp. By comparison with sequences in the public databases, 798 (60%) of the bandicoot ESTs had homology to known mammalian genes and were placed into several categories according to their putative biological functions (Figure 1). These included tissue specific transcripts encoding proteins involved in immune function and those that are ubiquitously expressed and involved in broad functions such as development and cell maintenance. A further 76 sequences (5.8%) matched previously identified coding regions of unknown function and 445 sequences (34%) resulted in no match with annotated sequences in any of the public databases (Figure 1).

### ESTs matching genes with known function

Figure 1 shows the distribution of transcripts identified in the bandicoot thymus. Not surprisingly, the most highly expressed transcripts were housekeeping genes necessary for cell maintenance within all tissues, including the thymus. As in many other EST studies, transcripts involved in protein metabolism were the most abundant (24%), followed by those involved in transcription and/or nucleic acid metabolism (10%) and metabolism/energy pathways (9%) (Figure 1). While many transcripts were identified only once (310 out of 798), 120 were detected between 2–22 times. A total of 430 different genes were identified among the 1319 clones analyzed, to our knowledge making it the largest current set of annotated genes for a Peramelid marsupial. Ribosomal proteins (assorted 40S and 60S) were the most redundant sequences identified, making up 221 ESTs (see Additional file 1). This is similar to the expression of ribosomal proteins in other EST libraries from the human thymus and other tissues [14,24]. Other highly expressed housekeeping genes encoded proteins necessary for intracellular protein trafficking, cell division and substance transport including cytochrome c oxidase (23 ESTs), histones (21 ESTs), NADH dehydrogenase (18 ESTs) and ubiquitin (15 ESTs) (see Additional file 1).

Since the function of the thymus is the maturation and selection of T cells, we were interested in the immune related ESTs among the bandicoot dataset. The thymus is important in the development of T cell tolerance to self major histocompatibility complex (MHC), including class Ib molecules with tissue specific distribution patterns such as those involved in fetal-maternal tolerance. Although the cDNA library used in the present study was from a juvenile male bandicoot, molecules involved in allorecognition are generally expressed in thymuses from both males and females, likely reflecting their role in immune regulation in addition to the fetal-maternal interface. The thymus therefore provides an overview of immune genes relevant to both general immunity and to mechanisms of immune tolerance.

Seventy of the 1319 ESTs showed homology to genes associated with immune function and/or T cell development (see Additional File 1). The expression profile of immune related genes in the bandicoot thymus is consistent with current knowledge of thymic T cell maturation in other mammals. T and B cell receptors and co-receptors, antigen processing genes, cytokine receptors and genes encoding molecules involved in innate immune function were among the immune related molecules identified among the bandicoot thymus EST dataset (Table 1).

The identification of immunoglobulin and CD79a transcripts indicates that B cells are present in the bandicoot thymus. This is consistent with previous reports indicating that B cells are present in the thymus of eutherians and marsupials. CD79a-positive cells have been observed in the thymuses of adult and developing marsupials [8,25,26]. In addition, the thymuses of neonatal and adult mice and adult humans contain a small population of B lymphocytes [27-29]. Nango et al. (1991) [29] have suggested that thymic B cells in humans may play a role in negative selection of T cells. It is likely their role is similar in marsupials.

Functional studies suggest that marsupials display some interesting differences in their immune response compared to eutherian mammals. For instance, marsupials are capable of rejecting allogenic skin grafts in a manner similar to that of eutherians, however, the average time for complete rejection appears to be significantly longer than the time observed for other mammalian species. In addition, weak or non-existent mixed lymphocyte responses and a poor capacity for immunoglobulin class switching have been reported for several marsupial species. Some marsupials also show increased susceptibility to pathogens such as *Mycobacterium tuberculosis *and *Trypansoma cruzi *(reviewed in [30]). Further information on the molecules involved in the marsupial immune response will assist in determining the mechanisms responsible for these differences.

A variety of immune related molecules not previously cloned from any marsupial were identified among the bandicoot ESTs. Amongst these were ESTs with homology to antigen processing genes including proteasome subunits (PSMB8, PSMB9, PSMB10 and PA28α) and the MHC associated class II invariant chain. Of the four cytokine receptor genes identified in the bandicoot thymus (TNFR, LTBR, IL-18R and IFNGR), only IFNGR has been previously identified in the whole genome sequence of *M. domestica *and none of these receptors have been cloned previously from a marsupial [31]. Four genes involved in the innate immune system were among the bandicoot thymus ESTs that have not been previously reported from a marsupial: complement component 3 (C3), proteoglycan core protein, glioma pathogenesis related protein 1 (GliPR1) and a homolog of a cathelicidin antimicrobial peptide (Table 1). C3 is the central component of the complement cascade important in innate immunity and inflammation. Homologs of complement components including C3 have been identified in a range of vertebrates and invertebrates providing evidence for an ancient origin of this system [32]. Proteoglycan core protein and GliPR1 are novel innate immune molecules not previously described in any marsupial. Proteoglycan core protein is the primary proteoglycan of cytotoxic granules and is involved in granule mediated apoptosis [33]. GliPR1 belongs to a group of plant pathogenesis-related proteins and is believed to play a role in the innate immune response of vertebrates [34]. The bandicoot cathelicidin is the first identified in any marsupial to date. Cathelicidins have been identified in a range of mammals and more recently in hagfish and rainbow trout [35,36]. Potential antimicrobial compounds have been identified in the pouches of koalas but none were homologous to known cathelicidins [37]. Such anti-microbial compounds likely provide an innate layer of protection for the vulnerable pouch-young prior to the development of their adaptive immune system. The identification of a variety of immune related molecules in the present study provides evidence that the complexity of the marsupial immune system is comparable to that of eutherian mammals despite the differences in immune responses previously reported.

### Comparison of bandicoot ESTs with the whole genome sequence of the opossum

The availability of a whole genome sequence from *M. domestica *allows for a significant comparison between two marsupial genomes. To better characterize the homology between the bandicoot EST sequences and the opossum genome, the 1319 bandicoot ESTs were clustered and assembled using EGassembler [38], resulting in 144 contigs and 805 singletons. Assembled ESTs were used for the cross species comparison to reduce the influence of high copy numbers of abundantly expressed transcripts in the raw EST dataset. Thirty EST sequences were discarded for being too short after masking and trimming. Contigs ranged in size from 254 to 1189 bp, with a median size of 546 bp. The largest cluster contained 11 transcripts and corresponds to 60S ribosomal protein L26.

Of the 949 assembled bandicoot ESTs, 797 (84%) found a match with sequence from the opossum genome (Table 2). By comparison, Rink et al. (2006) [39] reported that 1422 of 2035 pig ESTs (70%) found a match in the human genome. The divergence between artiodactyls and humans is estimated to have occurred around 80–100 MYA [40], similar to bandicoots and opossums which last shared a common ancestor approximately 75 MYA [40-42]. Given the time period separating bandicoots and opossums, the large proportion of bandicoot sequences that aligned with the opossum genome sequence is remarkable. The smaller dataset of bandicoot ESTs may have caused a bias towards the identification of highly expressed, highly conserved housekeeping genes, therefore resulting in a greater number of matches with sequence in the opossum genome sequence.

There were 152 assembled ESTs that did not align with the whole genome sequence of the opossum at either the nucleotide or amino acid level (Table 2). Not surprisingly, the majority of these ESTs (134) also had no hit with annotated sequences from the public databases and are included below in the analysis of non-coding RNAs (ncRNAs). Of the remaining 18, 15 matched proteins of known function and three matched sequences of unknown function in other species. It is possible that some of these genes are absent from the current opossum genome assembly due to unsequenced gaps. Alternatively, some genes may have diverged rapidly or been lost in the opossum lineage.

### Non-coding RNAs

Analyses of EST datasets from other species have reported a proportion of sequences that represent ncRNAs [43]. ncRNAs make up a significant proportion of the mammalian transcriptome and play a variety of roles, including transcriptional regulation, chromosome replication, RNA processing and modification, mRNA stability and translation, and protein degradation and translocation [44-46]. ncRNAs range from the 22 nucleotide family of microRNAs (miRNAs) to the 100–200 nucleotide small RNAs (sRNAs) commonly found as translational regulators in bacterial cells to >10000 nucleotides for RNAs involved in gene silencing in higher eukaryotes [46].

After assembly, the 445 ESTs that did not match an annotated sequence in the public databases yielded 375 unique sequences (consisting of 144 contigs and 231 singletons). These ranged from 106 to 1019 bp in length. Some of the shorter clones in this category may contain insufficient sequence to identify coding regions and many of these sequences may represent the 3' or 5' untranslated region (UTR) of genes. To determine whether open reading frames (ORFs) were present in any of the unmatched ESTs, the web-based program, ESTScan was used to scan these ESTs for potential ORFs [47]. This resulted in the identification of putative ORFs in only 14 of the 375 unmatched ESTs. These 14 ORFs ranged in length from 84–383 bp. It is possible that these sequences encode proteins that have not been identified in other species or they may be composed of sequences too divergent to be recognized by sequence comparison programs. To identify divergent gene homologs among the 375 unmatched ESTs, the six-frame translation was also searched using all profiles in the Pfam database using a hidden Markov model search program (HMMER), however no significant matches were identified.

**Figure 2:**
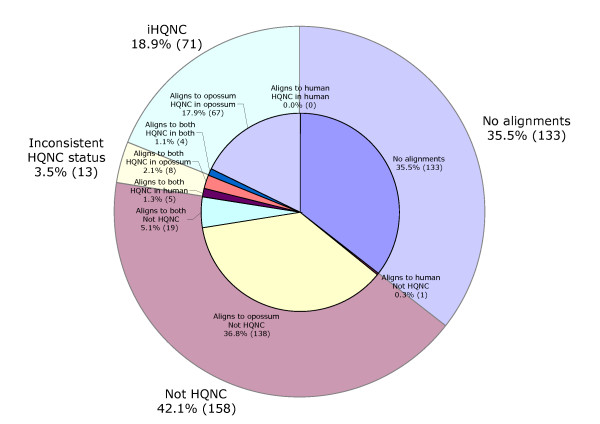
The distribution of results from aligning 375 unannotated assembled ESTs with the opossum and human genomes. An Integrated High Quality Non Coding (iHQNC) set of ESTs that aligned more than 5 kb from an annotated feature were identified. ESTs that aligned to HQNC regions in one genome but were within 5 kb of an annotated gene in the other genome are labeled "inconsistent" HQNC.

We investigated the possibility that some of the 375 unmatched bandicoot ESTs are conserved ncRNAs by comparing them to the opossum and human genome sequences (Figure 2; see Additional file 2) and their ENSEMBL or UCSC annotations. ESTs that aligned more than 5 kb from an annotated gene were labeled High Quality Non-Coding (HQNC) sequences. Of these 375 unmatched ESTs, 158 were considered unlikely ncRNA candidates because they either aligned to the opossum genome alone (138 clones) or the human genome alone (1 clone) or both (19 clones) and were within 5 kb of an annotated gene. These may be UTRs from coding mRNAs. Pre-miRNA and small nucleolar RNAs (snoRNAs) may also be represented in this group, as many miRNAs and snoRNAs are encoded in introns [48]. However, searching this set with the sequences of known pre-miRNAs and snoRNAs did not result in any significant matches (e-value ≤ 10^-4^). A further 13 ESTs aligned to both the human and opossum genomes, but were within 5 kb of an annotated gene in only one of the species. Thus, these ESTs obtained inconsistent HQNC status and are unlikely to be ncRNAs. Of the remaining unmatched ESTs, 133 aligned to neither the opossum nor human genomes, and are therefore not conserved. These may still be functional ncRNAs, since larger non-sno, non-micro ncRNAs (for example XIST and AIR) are poorly conserved and are thought to be under the influence of different evolutionary constraints [49]. The remaining 71 unmatched ESTs represent an integrated set of High Quality Non-Coding (iHQNC) sequences from comparison with two genomes and are therefore strong candidates for ncRNA. Of these 71 iHQNC sequences, 67 ESTs aligned to the opossum genome sequence only and may be marsupial specific ncRNAs (Fig. 2; see Additional file 2). Four ESTs aligned to both the human and opossum genomes and may represent ncRNAs conserved across all therian mammals. The function of both the marsupial specific and conserved ncRNAs identified in this study remains to be determined. However, since ncRNAs co-evolve with the genes they regulate it is not surprising that some are marsupial specific.

The 71 bandicoot iHQNC sequences represent 7.5% of the clustered and assembled EST dataset. By comparison, 7% of 60770 full length mouse cDNAs and 1.9% of a set of 19626 chicken EST contigs were identified as ncRNAs [43,45]. Thus, the results of the bandicoot EST analysis is comparable to that of the mouse and may be representative of the expression of ncRNAs in mammalian tissues.

## Conclusion

In summary, this study provides an overview of transcripts expressed in the bandicoot thymus, providing the opportunity for a genome wide comparison with the *M. domestica *whole genome sequence. Comparison of the bandicoot EST dataset with the opossum whole genome sequence revealed a high level of homology between the genomes of these two distantly related marsupials. This study has also expanded the number of immunity related genes known for marsupials, providing the first step in developing the tools necessary for further studies of immune function in marsupials. Overall, these results demonstrate the utility of an EST approach for gene discovery in marsupials and provide a useful resource of immune and non-immune related genes for transcriptional analysis in marsupials. Furthermore, the identification of genes specifically in the bandicoot that are likely to play a role in maternal-fetal interactions, will greatly enhance this species as a model organism. Such genes include those involved in allorecognition, such as genes of the MHC and TCR, as well as genes with a role in innate immunity, like the cathelicidin antimicrobial peptide.

## Methods

### Isolation and analysis of Expressed Sequence Tag (EST) clones

The thymus cDNA library and the isolation of ESTs have been described previously [50]. Briefly, two thousand randomly selected colonies were sequenced from a mass excision of the unamplified bandicoot thymus cDNA library using the T3 primer. Sequences were compared to sequences in the protein and nucleotide databases (Swissprot, nt and nr) in GenBank and the *M. domestica *whole genome sequence using the BLAST algorithms [51]. Only matches with e-values of ≤10^-5 ^were considered significant. Those sequences that consisted of vector sequence only, bad sequence reads or that were less than 100 nucleotides in length were excluded from the analysis. The web-based, ESTScan was used to scan sequences that showed no match with any of the public databases to identify whether they contained potential ORFs [47]. Sequences were categorized into groups according to their predicted biological function, following the classifications of The Gene Ontogeny Consortium (GO) [52] and human protein reference database [53]. The ESTs generated in this study have been submitted to GenBank (accession nos. EE743888–EE745206).

### Comparison with *M. domestica *genome sequence

To better characterize the homology between the bandicoot and the opossum, the ESTs were masked for mammalian repeats using RepeatMasker and then clustered and assembled using EGassembler [38]. Masking of repeats and organelle sequence was disabled in EGassembler.

The whole genome of the South American opossum, *M. domestica*, has been sequenced by the Broad Institute (MIT). All analyses were performed using MonDom4 (GenBank accession number AAFR03000000). The clustered and assembled ESTs were compared with the whole genome sequence of the opossum using BLASTN and TBLASTX [51]. Only matches with e-values ≤10^-4 ^were considered significant.

To identify diverged homologs in the unannotated ESTs, the 6 frame translation was searched with all Pfam profile hidden Markov models [54,55] using HMMer [56]. The unannotated ESTs were then aligned with predicted peptide sequences from the ENSEMBL *M. domestica *genebuild (Build 39) using BLASTX.

### Detection of conserved non-coding RNAs

To create a set of High Quality Non-Coding (HQNC) ESTs [43] we aligned the 375 ESTs that showed no match to annotated genes in the public database to the *M. domestica *genome (MonDom3, released January 2006) using BLASTN and TBLASTX. The best matches with e-values ≤ 10^-4 ^were compared with the Ensembl annotation (Build 39). Since UTRs are essentially unrepresented in this annotation, ESTs less than 5 kb from an ENSEMBL feature were considered to be possible UTRs. The remaining ESTs formed a HQNC set.

To make use of the higher quality annotation of the human genome, we also aligned the 375 unmatched ESTs to the human genome (NCBI Build 36.1, release March 2006). The best matches (e-value ≤ 10^-4^) were compared with the UCSC genome browser 'known gene' annotation (hg18), which is based on protein data from UniProt and mRNA data from the NCBI RefSeq collection and GenBank. Once again ESTs that were within 5 kb of an annotated gene were considered to be possible UTRs. This provided a second HQNC set based on a higher quality annotation, but using a more distantly related species.

The bandicoot HQNC ESTs identified from alignment to the opossum or human genomes were then combined into an integrated High Quality Non-coding (iHQNC) EST set. ESTs that aligned to both the opossum and human genomes and were members of the HQNC sets in both species were regarded as conserved HQNC and included in the iHQNC set. ESTs that aligned to only one genome and were present in the HQNC set for that species were also included as iHQNCs. ESTs that aligned to both genomes and were within 5 kb of an annotated gene in one of the species but not the other were regarded as having inconsistent HQNC status. The resulting iHQNC set thus contains conserved HQNC sequences and HQNC sequences that are highly diverged or putatively lost from one species or the other.

Finally, we compared the unmatched ESTs with known human snoRNAs from the snoRNA-LBME-db [57] and with predicted stem-loop sequences (which include the pre-miRNA) from miRBase [58,59] using BLASTN (e-value ≤ 10^-4^).

## Abbreviations

EST: Expressed sequence tag

HQNC: High quality non coding

iHQNC: Integrated high quality non coding

ORF: Open reading frame

MHC: Major Histocompatibility Complex

MYA: million years ago

miRNA: microRNA

ncRNA: non coding RNA

PSMB: Proteasome subunit beta

sRNA: small RNA

snoRNA: small nucleolar RNA

TCR: T cell receptor

UTR: Untranslated region

## Authors' contributions

MLB and RDM conceived the study, supervised data collection and analysis and wrote the manuscript. ATP participated in data analysis and manuscript preparation. GHR participated in data analysis and data management. SI and AMN participated in data collection. KL-T provided access to the whole genome database needed for analysis. All authors read and approved the final manuscript.

## Supplementary Material

Additional file 1 Summary of annotated Bandicoot ESTs. Table of bandicoot ESTs that match genes of known and unknown function in the public databases. Categories were assigned according to putative biological function using Gene Ontogeny and the Human Protein Reference Database [52,53]. The closest protein match to each EST according to BLASTx is indicated. Click here for file

Additional file 2 Summary of integrated High Quality Non Coding (iHQNC) and inconsistent HQNC ESTs. Table of unmatched bandicoot ESTs that aligned to the opossum or human genomes or both. ESTs that aligned to the opossum or human genome, or both and were more than 5 kb from an annotated gene were labeled iHQNC ESTs. Those ESTs that aligned to both the human and opossum genomes, but were more than 5 kb from an annotated gene in only one species were labeled inconsistent HQNC ESTs. Click here for file

## Figures and Tables

**Table 1:** Immunity related bandicoot ESTs

**Clone ID^a^**	**Gene**	**#**	**% Identity**	**Species Match**	**Positions^b^**	**Accession**
**T cell receptor complex**						
T013A03	TCRA	1	67	Rat	323–429	EE744980
T004C05	TCRB	12	90	Possum	15–167	EE744328
T005B12	TCRG	2	79	Tammar	33–92	EE744394
T005G01	TCRM*	6				EE744440
TImA207	CD3 epsilon	1	89	Tammar	107–189	EE745132
**Antigen processing genes**						
T011C11	MHC Class I	5	86	Possum	276–362	EE744882
T002B03	β2-microglobulin	5	92	Possum	21–122	EE745008
T002C02	MHC Class II	5	91	RN Wallaby	1–170	EE745063
T013D11	MHC Class II invariant chain (CD74)	2	77	Human	17–151	EE745023
T004A10	CD1	1	68	Human	12–83	EE744315
T010D03	BAT5	1	86	Cow	4–132	EE744822
TImA41	Proteasome activator complex	1	89	Mouse	115–249	EE743939
T005D01	Proteasome subunit beta type 9	1	84	Mouse	138–209	EE744406
TImA129	Proteasome subunit beta type 8	1	88	Human	249–273	EE745081
TImA698	Proteasome subunit beta type 10	2	98	Cow	104–277	EE744154
**B cell receptor complex**						
TImA501	Immunoglobulin heavy chain	2	84	Possum	278–397	EE744016
TImA494	Immunoglobulin lambda light chain	4	77	Possum	119–238	EE744009
TImA88	Immunoglobulin kappa light chain	1	86	Possum	16–146	EE744775
T010A10	CD79a	1	68	Mouse	28–133	EE744794
**Cytokine receptors**						
T012F10	Interleukin-18 receptor	1	57	Pig	17–97	EE744958
T002E04	Interferon-gamma receptor 1	2	57	Dog	372–466	EE745141
T008H12	Lymphotoxin beta receptor	1	65	Dog	223–362	EE744702
T013C12	Tumor necrosis factor receptor member	1	61	Dog	355–438	EE745012
**Innate immunity related genes**						
T005D05	Complement component 3	1	89	Tammar	1156–1651	EE744408
T004A09	Proteoglycan core protein	1	56	Chicken	1–96	EE744314
TImA656	Glioma pathogenesis protein	1	66	Human	150–214	EE744127
T007B06	Cathelicidin antimicrobial peptide	1	61	Dog	15–127	EE744556
**Signal transduction molecules involved in lymphocyte development and function**						
T010D12	CD45	1	49	Horse	1–81	EE744831
TImA137	CD37	1	65	Dog	24–70	EE745088
T008G06	CD53	1	65	Mouse	185–219	EE744685
T005F04	CD147	1	71	Cow	142–230	EE744430
T009G02	SH3 protein expressed in lymphocytes	1	79	Mouse	79–210	EE744765
T007G10	Src-like adaptor protein 2	1	69	Cow	160–273	EE744600
T005F01	NF-κB inhibitor beta	1	81	Dog	253–296	EE744428
**Function Unknown**						
T004E12	Interferon induced protein	1	72	Rat	9–141	EE744349

^a. ^Only clones that showed the lowest E value are shown for non-singletons

^b. ^Amino acid positions showing the highest similarity to the clone

* Previously described as a divergent TCRD [50].

**Table 2:** Comparison of 949 assembled bandicoot ESTs with the whole genome sequence of the opossum

**EST category**	**Match**	**Unmatched**
Immune	42	0
Apoptosis	5	0
Cell Cycle	9	0
Cell Growth and Maintenance	21	1
Cell Structure	12	0
Function Unknown	61	3
Metabolism	47	6
No Hit	241	134
Protein Metabolism	146	5
Signal Transduction	50	1
Transcription and/or Nucleic Acid Metabolism	100	1
Transport	63	1
**Total**	797	152
