# Supplementary material for: Analysis of a set of Australian northern brown bandicoot expressed sequence tags with comparison to the genome sequence of the South American grey short tailed opossum
Source: BMC Genomics. 2007 Feb 13;8:50. doi: 10.1186/1471-2164-8-50 (PMC1802078; doi:10.1186/1471-2164-8-50)
Supplement: Additional file 1 — Summary of annotated Bandicoot ESTs. Table of bandicoot ESTs that match genes of known and unknown function in the public databases. Categories were assigned according to putative biological function using Gene Ontogeny and the Human Protein Reference Database [52,53]. The closest protein match to each EST according to BLASTx is indicated. [file 1471-2164-8-50-S1.doc]

**Additional file 1. Summary of annotated Bandicoot ESTs**

| **Clone** | **Category** | **Protein Match** | **Accession** |
| --- | --- | --- | --- |
| T008D05 | Apoptosis | Apoptosis-associated speck-like protein containing a CARD | EE744650 |
| TImA380 | Apoptosis | Bcl10-interacting CARD protein | EE743910 |
| TImA71 | Apoptosis | Bcl10-interacting CARD protein | EE744165 |
| T006C05 | Apoptosis | Death associated protein 3 | EE744485 |
| T006A10 | Apoptosis | Death-associated protein 1 | EE744468 |
| T004E05 | Apoptosis | Defender against cell death 1 | EE744342 |
| T010D08 | Cell Cycle | Aurora kinase A-interacting protein | EE744827 |
| T004B12 | Cell Cycle | CDC45-related protein | EE744323 |
| T003F09 | Cell Cycle | Septin-1 | EE744284 |
| T009E04 | Cell Cycle | Septin-1 | EE744743 |
| T001C08 | Cell Cycle | Septin-9 | EE744387 |
| T004A03 | Cell Cycle | Similar to RAB GTPase activating protein isoform 5 | EE744308 |
| T005G02 | Cell Cycle | Sperm-associated antigen 5 | EE744441 |
| T006A12 | Cell Cycle | Transcription factor E2F5 | EE744470 |
| TImA556 | Cell Cycle | Tumor suppressor candidate 4 | EE744069 |
| T008B04 | Cell Growth and Maintenance | Actin cytoplasmic 1 | EE744625 |
| T008A04 | Cell Growth and Maintenance | Actin regulatory protein | EE744613 |
| T010G06 | Cell Growth and Maintenance | Actin regulatory protein | EE744855 |
| T001B11 | Cell Growth and Maintenance | Actin-related protein 2/3 complex subunit 4 | EE744310 |
| TImA711 | Cell Growth and Maintenance | B-cell translocation gene 1 | EE744168 |
| T006F09 | Cell Growth and Maintenance | clathrin heavy polypeptide | EE744523 |
| T001E02 | Cell Growth and Maintenance | Coactosin-like protein | EE744542 |
| T008C02 | Cell Growth and Maintenance | Coronin-1A | EE744635 |
| TImA75 | Cell Growth and Maintenance | Coronin-1A | EE744204 |
| T005E10 | Cell Growth and Maintenance | Coronin-1A | EE744425 |
| T007C11 | Cell Growth and Maintenance | Coronin-1A | EE744569 |
| TImA79 | Cell Growth and Maintenance | Dickkopf-like protein 1 | EE744235 |
| T001B07 | Cell Growth and Maintenance | Dynein heavy chain | EE744277 |
| TImA118 | Cell Growth and Maintenance | Ezrin | EE745076 |
| T008H09 | Cell Growth and Maintenance | Ezrin | EE744699 |
| T001E05 | Cell Growth and Maintenance | F-actin capping protein alpha-2 | EE744564 |
| T001D11 | Cell Growth and Maintenance | Fascin | EE744508 |
| TImA790 | Cell Growth and Maintenance | Fibronectin type III domain containing 3A | EE744236 |
| T003F05 | Cell Growth and Maintenance | Gelsolin precursor | EE744281 |
| T008G01 | Cell Growth and Maintenance | Keratin, type I cytoskeletal 18 | EE744680 |
| T006D11 | Cell Growth and Maintenance | Keratin, type II cytoskeletal 6A | EE744503 |
| T008C06 | Cell Growth and Maintenance | Keratin, type II cytoskeletal 8 | EE744638 |
| TImA636 | Cell Growth and Maintenance | Kinesin family member 17 | EE744116 |
| T010C10 | Cell Growth and Maintenance | microtubule associated protein like 4 | EE744816 |
| TImA393 | Cell Growth and Maintenance | Microtubule associated protein RP/EB family | EE743923 |
| T013B05 | Cell Growth and Maintenance | Myosin light polypeptide 6 | EE744993 |
| TImA437 | Cell Growth and Maintenance | Myosin light polypeptide 6 | EE743964 |
| T008C10 | Cell Growth and Maintenance | Nesprin-1 | EE744643 |
| T007F08 | Cell Growth and Maintenance | Nuclear mitotic apparatus protein 1 | EE744589 |
| TImA504 | Cell Growth and Maintenance | oxidation resistance 1 isoform 1 | EE744018 |
| T005D07 | Cell Growth and Maintenance | p53 inducible protein PIR121 | EE744411 |
| T012B09 | Cell Growth and Maintenance | senescence-associated protein | EE744923 |
| T003E08 | Cell Growth and Maintenance | similar to C1q domain containing 1 isoform 1 isoform 9 | EE744274 |
| TImA595 | Cell Growth and Maintenance | similar to testes development-related NYD-SP20 | EE744092 |
| T008G04 | Cell Growth and Maintenance | similar to thymosin, beta 4 | EE744683 |
| T003G04 | Cell Growth and Maintenance | Small EDRK-rich factor 2 | EE744290 |
| T013A12 | Cell Growth and Maintenance | stromal membrane-associated protein | EE744989 |
| TImA201 | Cell Growth and Maintenance | thymosin beta 4 | EE745126 |
| T006B02 | Cell Growth and Maintenance | Thymosin beta-4 | EE744472 |
| T006E09 | Cell Growth and Maintenance | Thymosin beta-4 | EE744512 |
| T001B05 | Cell Growth and Maintenance | thymosin-like 4 | EE744266 |
| T006E04 | Cell Growth and Maintenance | Translationally controlled tumor protein | EE744507 |
| T008H10 | Cell Growth and Maintenance | Translationally controlled tumor protein | EE744700 |
| T005G05 | Cell Growth and Maintenance | Tubulin alpha chain | EE744444 |
| TImA709 | Function Unknown | 6.8 kDa mitochondrial proteolipid | EE744164 |
| TImA624 | Function Unknown | ankyrin repeat domain | EE744109 |
| T005D10 | Function Unknown | ARMET protein precursor | EE744414 |
| T005A06 | Function Unknown | BHD protein | EE744379 |
| T013H09 | Function Unknown | Breast carcinoma amplified sequence 2 | EE745062 |
| T010D06 | Function Unknown | CG7506-PA | EE744825 |
| T005A08 | Function Unknown | Chromatin-associated protein Dek and related proteins | EE744381 |
| T010D07 | Function Unknown | chromosomal protein | EE744826 |
| T004C11 | Function Unknown | conserved alpha-helical protein | EE744330 |
| T007G05 | Function Unknown | Cricetulus griseus external transcribed spacer | EE744596 |
| T010A03 | Function Unknown | DNA sequence from clone XX-NCIH2171 | EE744788 |
| TImA295 | Function Unknown | Extensin-3 precurosr | EE745194 |
| TImA481 | Function Unknown | glycine rich protein | EE743997 |
| T001D10 | Function Unknown | HISTIDINE-RICH GLYCOPROTEIN | EE744497 |
| T003A10 | Function Unknown | hypothetical protein | EE744066 |
| T003G01 | Function Unknown | Hypothetical protein | EE744287 |
| T005B07 | Function Unknown | Hypothetical protein | EE744389 |
| T005F12 | Function Unknown | Hypothetical protein | EE744439 |
| T006D02 | Function Unknown | Hypothetical protein | EE744495 |
| T006F01 | Function Unknown | Hypothetical protein | EE744515 |
| T007E07 | Function Unknown | hypothetical protein | EE744582 |
| T008A10 | Function Unknown | hypothetical protein | EE744620 |
| T009C05 | Function Unknown | hypothetical protein | EE744721 |
| T009G08 | Function Unknown | hypothetical protein | EE744771 |
| T010A06 | Function Unknown | hypothetical protein | EE744790 |
| T011A11 | Function Unknown | hypothetical protein | EE744871 |
| T012B11 | Function Unknown | hypothetical protein | EE744925 |
| T012D02 | Function Unknown | hypothetical protein | EE744938 |
| T012F06 | Function Unknown | hypothetical protein | EE744954 |
| T013F07 | Function Unknown | hypothetical protein | EE745039 |
| T008F04 | Function Unknown | hypothetical protein | EE744672 |
| TImA289 | Function Unknown | hypothetical protein | EE745188 |
| TImA292 | Function Unknown | hypothetical protein | EE745192 |
| TImA613 | Function Unknown | hypothetical protein | EE744104 |
| T013F04 | Function Unknown | Hypothetical Protein KIAA0562 | EE745038 |
| T009E05 | Function Unknown | Hypothetical protein LOC221143 | EE744744 |
| T009G06 | Function Unknown | Hypothetical protein UPF0195 | EE744769 |
| T006A11 | Function Unknown | Iron sulfur assembly protein 1 | EE744469 |
| T007A06 | Function Unknown | KDEL (Lys-Asp-Glu-Leu) containing 1 | EE744546 |
| T003D05 | Function Unknown | KIAA0674 | EE744261 |
| T006E12 | Function Unknown | KIAA0885 protein | EE744514 |
| T004G04 | Function Unknown | KIAA1109 hypothetical protein | EE744361 |
| T009C02 | Function Unknown | KIAA1731 protein | EE744718 |
| TImA143 | Function Unknown | KIAA2010 protein | EE745093 |
| T012F04 | Function Unknown | Lectin | EE744951 |
| T004F12 | Function Unknown | metadherin (MTDH), mRNA | EE744357 |
| T005H06 | Function Unknown | metallophosphoesterase domain containing 2, mRNA | EE744456 |
| T001D05 | Function Unknown | NIPA-like domain containing 3 | EE744442 |
| T002A02 | Function Unknown | novel protein | EE744930 |
| T007H09 | Function Unknown | NS1A protein | EE744607 |
| T003D01 | Function Unknown | Nuclear domain 10 protein | EE744257 |
| T006F11 | Function Unknown | phosphoglycerate kinase | EE744524 |
| T005H02 | Function Unknown | Predicted membrane protein | EE744452 |
| T008H07 | Function Unknown | PREDICTED: similar to B5 receptor | EE744696 |
| TImA426 | Function Unknown | Protein C14orf166 | EE743952 |
| T006D06 | Function Unknown | Protein C21orf51 homolog | EE744500 |
| T002A03 | Function Unknown | Protein C7orf24 homolog | EE744941 |
| T010A02 | Function Unknown | Protein CHMP7 | EE744787 |
| T013D08 | Function Unknown | Protein CXorf33 homolog precursor | EE745020 |
| T001F02 | Function Unknown | Protein KIAA1115 | EE744630 |
| T003A07 | Function Unknown | putative nucleic acid binding protein RY-1 | EE744044 |
| T010C05 | Function Unknown | RIKEN cDNA 1810014F10 | EE744812 |
| TImA635 | Function Unknown | sel-1 suppressor of lin-12 like | EE744115 |
| TImA290 | Function Unknown | SH2 domain ptotein 2A | EE745190 |
| TImA489 | Function Unknown | similar to C01G5.8 isoform 3 | EE744007 |
| T002B02 | Function Unknown | similar to CG12025-PA isoform 1 | EE744997 |
| T007B07 | Function Unknown | Similar to FLJ35382 protein | EE744557 |
| TImA87 | Function Unknown | similar to KIAA1018 protein | EE744241 |
| T008H03 | Function Unknown | Transcription factor GT-2 | EE744692 |
| TImA291 | Function Unknown | U1 snRNP specific protein C | EE745191 |
| T008G05 | Function Unknown | unnamed protein product | EE744684 |
| T010A11 | Function Unknown | UPF0347 protein LOC55831 homolog | EE744795 |
| TImA73 | Function Unknown | WD-repeat protein 22 | EE744184 |
| T009E08 | Function Unknown | WD-repeat protein 46 | EE744747 |
| TImA405 | Function Unknown | WD-repeat protein 53 | EE743935 |
| T013H06 | Function Unknown | Wdr45 like | EE745060 |
| T007B06 | Immune | Antibacterial protein FALL-39 precursor | EE744556 |
| T010D03 | Immune | BAT5 (HLA-B associated transcript 5) | EE744822 |
| TImA32 | Immune | Beta-2-microglobulin | EE745203 |
| T001F06 | Immune | Beta-2-microglobulin precursor | EE744664 |
| T002B03 | Immune | Beta-2-microglobulin precursor | EE745008 |
| T010G08 | Immune | Beta-2-microglobulin precursor | EE744857 |
| T013H02 | Immune | Beta-2-microglobulin precursor | EE745056 |
| T005F04 | Immune | CD147 | EE744430 |
| T004A10 | Immune | CD1a antigen | EE744315 |
| T010D12 | Immune | CD45 leukocyte common antigen | EE744831 |
| T010A10 | Immune | CD79a | EE744794 |
| T005D05 | Immune | Complement C3 precursor | EE744408 |
| TImA436 | Immune | T cell receptor | EE743963 |
| TImA656 | Immune | Glioma pathogenesis-related protein 1 precursor | EE744127 |
| TImA88 | Immune | Ig kappa | EE744775 |
| TImA501 | Immune | immunoglobulin alpha heavy chain constant region | EE744016 |
| T006A09 | Immune | immunoglobulin heavy chain V region | EE744467 |
| T005E11 | Immune | immunoglobulin lambda light chain | EE744426 |
| TImA494 | Immune | immunoglobulin lambda light chain | EE744009 |
| TImA533 | Immune | immunoglobulin lambda light chain | EE744046 |
| T006F12 | Immune | immunoglobulin lambda light chain variable region | EE744525 |
| T001G02 | Immune | Interferon-gamma receptor alpha chain precursor | EE744741 |
| T002E04 | Immune | Interferon-gamma receptor alpha chain precursor | EE745141 |
| T004E12 | Immune | Interferon-induced 35 kDa protein | EE744349 |
| T012F10 | Immune | Interleukin-18 receptor 1 precursor | EE744958 |
| TImA137 | Immune | Leukocyte antigen CD37 | EE745088 |
| T008G06 | Immune | Leukocyte surface antigen CD53 | EE744685 |
| T008H12 | Immune | Lymphotoxin beta receptor | EE744702 |
| T001A12 | Immune | MHC class I | EE744110 |
| T011C03 | Immune | MHC class I | EE744880 |
| T011C11 | Immune | MHC class I | EE744882 |
| TImA557 | Immune | MHC class I | EE744070 |
| T002E01 | Immune | MHC class I antigen | EE745119 |
| T009G09 | Immune | MHC class II | EE744772 |
| T009G10 | Immune | MHC class II | EE744773 |
| TImA264 | Immune | MHC class II | EE745164 |
| TImA283 | Immune | MHC class II alpha chain | EE745182 |
| T011E04 | Immune | MHC class II associated invariant chain | EE744890 |
| T002C02 | Immune | MHC class II DR alpha protein precursor | EE745063 |
| T013D11 | Immune | MHC class II, gamma chain | EE745023 |
| T005F01 | Immune | NF-kappaB inhibitor beta | EE744428 |
| T004A09 | Immune | Platelet proteoglycan core protein | EE744314 |
| TImA41 | Immune | Proteasome activator complex subunit 1 | EE743939 |
| TImA454 | Immune | Proteasome subunit beta type 10 | EE743979 |
| TImA698 | Immune | Proteasome subunit beta type 7 | EE744154 |
| T005D01 | Immune | PSMB9 | EE744406 |
| T009G02 | Immune | SH3 protein expressed in lymphocytes | EE744765 |
| T007G10 | Immune | Src-like adaptor 2 | EE744600 |
| T013A03 | Immune | T cell receptor alpha chain V region | EE744980 |
| T004C05 | Immune | T cell receptor beta chain | EE744328 |
| T005A01 | Immune | T cell receptor beta chain | EE744375 |
| T008D07 | Immune | T cell receptor beta chain | EE744654 |
| TImA157 | Immune | T cell receptor beta chain | EE745103 |
| TImA446 | Immune | T cell receptor beta chain | EE743973 |
| TImA713 | Immune | T cell receptor beta chain | EE744170 |
| TImA730 | Immune | T cell receptor beta chain | EE744185 |
| T007C03 | Immune | T cell receptor beta chain | EE744562 |
| TImA407 | Immune | T cell receptor beta chain | EE743936 |
| TImA313 | Immune | T cell receptor beta chain C region | EE745200 |
| T013G09 | Immune | T cell receptor beta chain V region | EE745051 |
| T001G03 | Immune | T cell receptor delta | EE744752 |
| TImA125 | Immune | T cell receptor delta | EE745080 |
| T005G01 | Immune | T cell receptor delta-chain | EE744440 |
| T005B12 | Immune | T cell receptor gamma chain | EE744394 |
| T001B03 | Immune | T cell receptor gamma chain V region | EE744221 |
| TImA485 | Immune | T-cell receptor beta chain constant region | EE744003 |
| TImA207 | Immune | T-cell surface glycoprotein CD3 epsilon chain precursor | EE745132 |
| T002E03 | Immune | TCRM | EE745130 |
| T007F10 | Immune | TCRM | EE744591 |
| T013C12 | Immune | Tumor necrosis factor receptor superfamily member | EE745012 |
| T009E09 | Metabolism | 3-hydroxyacyl-CoA dehydrogenase type II | EE744748 |
| T009E10 | Metabolism | 3-hydroxyacyl-CoA dehydrogenase type II | EE744749 |
| T008F02 | Metabolism | 4F2 cell-surface antigen heavy chain | EE744670 |
| T012C08 | Metabolism | Acetylcholinesterase precursor | EE744933 |
| T002D02 | Metabolism | Acyl-CoA dehydrogenase | EE745108 |
| TImA558 | Metabolism | Asparaginyl-tRNA synthetase | EE744071 |
| T006B04 | Metabolism | ATP synthase a chain | EE744473 |
| TImA267 | Metabolism | ATP synthase a chain | EE745166 |
| TImA505 | Metabolism | ATP synthase a chain | EE744019 |
| TImA68 | Metabolism | ATP synthase a chain | EE744143 |
| T009G01 | Metabolism | ATP synthase gamma chain | EE744762 |
| T006B06 | Metabolism | ATP synthase lipid-binding protein | EE744476 |
| T007A03 | Metabolism | ATP synthase lipid-binding protein | EE744544 |
| T007E04 | Metabolism | ATP synthase lipid-binding protein | EE744579 |
| TImA596 | Metabolism | ATP synthase lipid-binding protein | EE744093 |
| T012B01 | Metabolism | Chitinase-like protein Idgf1 precursor | EE744917 |
| T001G10 | Metabolism | Cytochrome b | EE744819 |
| TImA743 | Metabolism | Cytochrome b | EE744196 |
| TImA495 | Metabolism | Cytochrome b | EE744010 |
| TImA539 | Metabolism | Cytochrome b | EE744051 |
| TImA679 | Metabolism | Cytochrome b | EE744142 |
| TImA712 | Metabolism | Cytochrome c | EE744169 |
| T012C02 | Metabolism | cytochrome c oxidase | EE744928 |
| T012C03 | Metabolism | cytochrome c oxidase | EE744929 |
| T006D10 | Metabolism | Cytochrome c oxidase polypeptide Va | EE744502 |
| T010B08 | Metabolism | Cytochrome c oxidase polypeptide Vb | EE744804 |
| TImA205 | Metabolism | Cytochrome c oxidase polypeptide Vb | EE745129 |
| T003D10 | Metabolism | Cytochrome c oxidase polypeptide VIc precursor | EE744265 |
| T009C09 | Metabolism | Cytochrome c oxidase polypeptide VIIb | EE744725 |
| T009C10 | Metabolism | Cytochrome c oxidase polypeptide VIIb | EE744726 |
| T001C05 | Metabolism | Cytochrome c oxidase subunit 1 | EE744354 |
| T007H05 | Metabolism | Cytochrome c oxidase subunit 1 | EE744604 |
| T010D04 | Metabolism | Cytochrome c oxidase subunit 1 | EE744823 |
| T012C01 | Metabolism | Cytochrome c oxidase subunit 1 | EE744927 |
| T013H01 | Metabolism | Cytochrome c oxidase subunit 1 | EE745055 |
| TImA124 | Metabolism | Cytochrome c oxidase subunit 1 | EE745079 |
| T008G07 | Metabolism | Cytochrome c oxidase subunit 1 | EE744687 |
| TImA646 | Metabolism | Cytochrome c oxidase subunit 1 | EE744123 |
| TImA50 | Metabolism | Cytochrome c oxidase subunit 1 | EE744014 |
| T012E02 | Metabolism | Cytochrome c oxidase subunit 1 | EE744944 |
| T012H05 | Metabolism | Cytochrome c oxidase subunit 1 | EE744970 |
| T006C07 | Metabolism | Cytochrome c oxidase subunit 2 | EE744488 |
| TImA274 | Metabolism | Cytochrome c oxidase subunit 2 | EE745172 |
| TImA275 | Metabolism | Cytochrome c oxidase subunit 2 | EE745173 |
| TImA772 | Metabolism | Di-N-acetylchitobiase precursor | EE744220 |
| T013G05 | Metabolism | Diamine acetyltransferase 1 | EE745048 |
| T010G05 | Metabolism | Dolichol-phosphate mannosyltransferase subunit 3 | EE744854 |
| T011D11 | Metabolism | Electron transfer flavoprotein beta-subunit | EE744888 |
| TImA543 | Metabolism | Electron transfer flavoprotein beta-subunit | EE744057 |
| T009A12 | Metabolism | ferrochelatase | EE744710 |
| T009F09 | Metabolism | ferrochelatase | EE744759 |
| T009F10 | Metabolism | ferrochelatase | EE744760 |
| T001D04 | Metabolism | Galactose-3-O-sulfotransferase 4 | EE744431 |
| T013B01 | Metabolism | Gamma-interferon-inducible protein IP-30 | EE744990 |
| TImA738 | Metabolism | Glutathione S-transferase theta 2 | EE744192 |
| TImA740 | Metabolism | Glutathione S-transferase theta 2 | EE744194 |
| T009A08 | Metabolism | Glutathione S-transferase theta 2 | EE744707 |
| T012G07 | Metabolism | Glyceraldehyde-3-phosphate dehydrogenase | EE744962 |
| T008H06 | Metabolism | Glyceraldehyde-3-phosphate dehydrogenase | EE744695 |
| T001B04 | Metabolism | Heme oxygenase 2 | EE744255 |
| T001D03 | Metabolism | Histone acetyltransferase-1 | EE744420 |
| T002A08 | Metabolism | Hydroxyacylglutathione hydrolase | EE744986 |
| T007B11 | Metabolism | hypoxanthine phosphoribosyltransferase | EE744560 |
| TImA616 | Metabolism | Inner membrane protein OXA1L | EE744106 |
| T005E09 | Metabolism | Ketohexokinase | EE744424 |
| T012C12 | Metabolism | L-lactate dehydrogenase B chain | EE744936 |
| T001F01 | Metabolism | Low-density lipoprotein receptor precursor | EE744619 |
| T008C03 | Metabolism | Lung carbonyl reductase | EE744636 |
| T012A08 | Metabolism | Lysosomal acid phosphatase precursor | EE744914 |
| T003A04 | Metabolism | Lysosomal thiol reductase | EE744022 |
| T009F06 | Metabolism | major facilitator superfamily domain containing 1 | EE744756 |
| TImA253 | Metabolism | mesoderm specific transcript homolog | EE745158 |
| T006A08 | Metabolism | methionine adenosyltransferase II, alpha | EE744466 |
| TImA122 | Metabolism | methyltransferase-like 2 | EE745078 |
| T004D04 | Metabolism | Mitochondrial associated endoribonuclease MAR1 | EE744333 |
| T005D08 | Metabolism | N-terminal acetyltransferase complex ARD1 subunit homolog | EE744412 |
| T013C04 | Metabolism | NADH dehydrogenase subunit 3 | EE745005 |
| T001C07 | Metabolism | NADH ubiquinone oxidoreductase | EE744376 |
| T008B10 | Metabolism | NADH ubiquinone oxidoreductase chain 4 | EE744632 |
| TImA357 | Metabolism | NADH-ubiquinone oxidoreductase | EE743899 |
| T002G06 | Metabolism | NADH-ubiquinone oxidoreductase | EE743933 |
| T005G09 | Metabolism | NADH-ubiquinone oxidoreductase 49 kDa subunit, mitochondrial precursor | EE744448 |
| TImA413 | Metabolism | NADH-ubiquinone oxidoreductase B9 subunit | EE743942 |
| T005C06 | Metabolism | NADH-ubiquinone oxidoreductase chain 1 | EE744400 |
| T001F09 | Metabolism | NADH-ubiquinone oxidoreductase chain 1 | EE744686 |
| TImA570 | Metabolism | NADH-ubiquinone oxidoreductase chain 2 | EE744081 |
| T009B09 | Metabolism | NADH-ubiquinone oxidoreductase chain 4 | EE744715 |
| T009B10 | Metabolism | NADH-ubiquinone oxidoreductase chain 4 | EE744716 |
| T013G06 | Metabolism | NADH-ubiquinone oxidoreductase chain 4 | EE745049 |
| TImA451 | Metabolism | NADH-ubiquinone oxidoreductase chain 4L | EE743975 |
| T010F07 | Metabolism | NADH-ubiquinone oxidoreductase MLRQ subunit | EE744846 |
| TImA530 | Metabolism | NADH-ubiquinone oxidoreductase MWFE subunit | EE744042 |
| TImA690 | Metabolism | NADH-ubiquinone oxidoreductase SGDH subun | EE744149 |
| TImA466 | Metabolism | NADH-ubiquinone oxidoreductase SGDH subunit, mitochondrial precursor | EE743987 |
| TImA119 | Metabolism | NADP-dependent malic enzyme, mitochondrial precursor | EE745077 |
| T007C04 | Metabolism | Ornithine decarboxylase antizyme | EE744563 |
| T011H06 | Metabolism | Ornithine decarboxylase antizyme | EE744905 |
| TImA255 | Metabolism | ORNITHINE DECARBOXYLASE ANTIZYME | EE745160 |
| T009C08 | Metabolism | Ornithine decarboxylase antizyme | EE744724 |
| T004B04 | Metabolism | Ornithine decarboxylase antizyme (ODC-Az) | EE744319 |
| T008D04 | Metabolism | Peroxiredoxin 2 | EE744649 |
| T011H07 | Metabolism | Peroxiredoxin 6 | EE744906 |
| TImA323 | Metabolism | phosphoglycerate kinase | EE745206 |
| TImA580 | Metabolism | phosphoglycerate kinase | EE744086 |
| T010H10 | Metabolism | Phospholipid transfer protein precursor | EE744865 |
| T007C08 | Metabolism | Phytanoyl-CoA dioxygenase | EE744566 |
| TImA400 | Metabolism | Protein N-terminal asparagine amidohydrolase | EE743929 |
| T012G11 | Metabolism | Pyruvate dehydrogenase protein X component, mitochondrial precursor | EE744967 |
| T012H07 | Metabolism | Quinone oxidoreductase | EE744972 |
| T012B03 | Metabolism | squalene epoxidase | EE744920 |
| T010B03 | Metabolism | Synaptic glycoprotein SC2 | EE744800 |
| T010G04 | Metabolism | Transaldolase | EE744853 |
| T005G07 | Metabolism | Transketolase | EE744446 |
| T002A04 | Metabolism | Valosin-containing protein | EE744952 |
| T010C09 | Nucleic Acid Metabolism | Activator 1 37 kDa subunit | EE744815 |
| T006E11 | Nucleic Acid Metabolism | Ankyrin repeat domain protein 11 | EE744513 |
| TImA21 | Nucleic Acid Metabolism | arginine/serine-rich 1 splicing factor | EE745135 |
| T012G12 | Nucleic Acid Metabolism | calmodulin binding transcription activator 2 | EE744968 |
| TImA146 | Nucleic Acid Metabolism | Chromodomain helicase-DNA-binding protein 3 | EE745095 |
| T010B10 | Nucleic Acid Metabolism | Cold-inducible RNA-binding protein | EE744806 |
| T002B04 | Nucleic Acid Metabolism | Core histone macro-H2A.1 | EE745019 |
| T010F03 | Nucleic Acid Metabolism | Core histone macro-H2A.1 | EE744842 |
| T013G07 | Nucleic Acid Metabolism | Core histone macro-H2A.1 | EE745050 |
| TImA99 | Nucleic Acid Metabolism | DEAD-box protein p68 | EE744245 |
| T010B01 | Nucleic Acid Metabolism | DNA binding protein | EE744798 |
| T013C10 | Nucleic Acid Metabolism | DNA binding protein | EE745010 |
| T010F10 | Nucleic Acid Metabolism | DNA polymerase alpha | EE744848 |
| T004G08 | Nucleic Acid Metabolism | DNA polymerase epsilon | EE744364 |
| T003E02 | Nucleic Acid Metabolism | DNA replication licensing factor MCM5 | EE744269 |
| T013E07 | Nucleic Acid Metabolism | DNA topoisomerase 2-beta | EE745029 |
| T011E11 | Nucleic Acid Metabolism | DNA topoisomerase I | EE744892 |
| T009A11 | Nucleic Acid Metabolism | E1A-associated protein p300 | EE744709 |
| T011B08 | Nucleic Acid Metabolism | Eukaryotic translation initiation factor 3 subunit 6 | EE744877 |
| T010F09 | Nucleic Acid Metabolism | Eukaryotic translation initiation factor 4H | EE744847 |
| T002B06 | Nucleic Acid Metabolism | Fanconi anemia, complementation group E | EE745030 |
| TImA168 | Nucleic Acid Metabolism | H2A histone family | EE745110 |
| TImA354 | Nucleic Acid Metabolism | H2A histone family, member Y isoform 2 | EE743898 |
| TImA518 | Nucleic Acid Metabolism | H2A histone family, member Z | EE744031 |
| TImA756 | Nucleic Acid Metabolism | H3 histone | EE744207 |
| TImA114 | Nucleic Acid Metabolism | H3 histone | EE745072 |
| TImA720 | Nucleic Acid Metabolism | H3 histone | EE744179 |
| TImA532 | Nucleic Acid Metabolism | H3 histone, family 3A | EE744045 |
| T006F04 | Nucleic Acid Metabolism | Heterogeneous nuclear ribonucleoprotein A1 | EE744517 |
| T013D09 | Nucleic Acid Metabolism | Heterogeneous nuclear ribonucleoproteins C1/C2 | EE745021 |
| TImA779 | Nucleic Acid Metabolism | High mobility group box 1 | EE744225 |
| TImA794 | Nucleic Acid Metabolism | High mobility group box 1 | EE744238 |
| T011A08 | Nucleic Acid Metabolism | High mobility group box 1 | EE744869 |
| T012B10 | Nucleic Acid Metabolism | High mobility group box 1 | EE744924 |
| T012F07 | Nucleic Acid Metabolism | High mobility group protein 1 | EE744955 |
| TImA398 | Nucleic Acid Metabolism | High mobility group protein 1 | EE743927 |
| T005C11 | Nucleic Acid Metabolism | High mobility group protein 2 | EE744404 |
| TImA498 | Nucleic Acid Metabolism | High mobility group protein 2 | EE744013 |
| TImA72 | Nucleic Acid Metabolism | histone acetyltransferase | EE744178 |
| T004D05 | Nucleic Acid Metabolism | Histone chaperone cia1 | EE744334 |
| T004F04 | Nucleic Acid Metabolism | Histone deacetylase complex subunit SAP18 | EE744351 |
| T013D05 | Nucleic Acid Metabolism | Histone deacetylase complex subunit SAP18 | EE745016 |
| T008G10 | Nucleic Acid Metabolism | Histone family, member Z | EE744689 |
| T009D11 | Nucleic Acid Metabolism | Histone H2A.z | EE744739 |
| T012A12 | Nucleic Acid Metabolism | Histone H2A.z | EE744916 |
| TImA444 | Nucleic Acid Metabolism | Histone H2A.z | EE743971 |
| T011D05 | Nucleic Acid Metabolism | Histone H3 | EE744884 |
| TImA430 | Nucleic Acid Metabolism | Histone H3.2 | EE743958 |
| T001G06 | Nucleic Acid Metabolism | Histone H3.3 | EE744786 |
| T004D11 | Nucleic Acid Metabolism | Histone H3.3 | EE744338 |
| T004F05 | Nucleic Acid Metabolism | Histone H3.3 | EE744352 |
| T009B01 | Nucleic Acid Metabolism | Histone H3.3 | EE744711 |
| T009G05 | Nucleic Acid Metabolism | Histone H3.3 | EE744768 |
| T008E07 | Nucleic Acid Metabolism | Histone H3.3 | EE744663 |
| TImA281 | Nucleic Acid Metabolism | Histone H3.3 | EE745180 |
| TImA376 | Nucleic Acid Metabolism | Histone H3.3 | EE743908 |
| TImA471 | Nucleic Acid Metabolism | Histone H3.3 | EE743990 |
| TImA488 | Nucleic Acid Metabolism | Histone H3.3 | EE744006 |
| T009E06 | Nucleic Acid Metabolism | Histone-lysine N-methyltransferase | EE744745 |
| T008A09 | Nucleic Acid Metabolism | Homeobox protein Meis2 | EE744618 |
| T010B06 | Nucleic Acid Metabolism | KH domain containing | EE744802 |
| TImA508 | Nucleic Acid Metabolism | leucine zipper and W2 domains 1 | EE744021 |
| T010C11 | Nucleic Acid Metabolism | LINE-1 reverse transcriptase homolog | EE744817 |
| TImA351 | Nucleic Acid Metabolism | Mago nashi protein homolog | EE743895 |
| T005F07 | Nucleic Acid Metabolism | mRNA turnover protein 4 homolog | EE744434 |
| T010B07 | Nucleic Acid Metabolism | MTG8 llike protein | EE744803 |
| T009D07 | Nucleic Acid Metabolism | N2,N2-dimethylguanosine tRNA methyltransferase | EE744735 |
| T003D02 | Nucleic Acid Metabolism | NIF3-like protein 1 | EE744258 |
| TImA606 | Nucleic Acid Metabolism | nuclear ribonucleoprotein K | EE744101 |
| T013H10 | Nucleic Acid Metabolism | Nuclear RNA export factor 1 | EE745064 |
| T008E01 | Nucleic Acid Metabolism | Nuclear transcription factor Y subunit gamma | EE744658 |
| T010G02 | Nucleic Acid Metabolism | Nuclear ubiquitous casein and cyclin-dependent kinases substrate (P1) | EE744850 |
| TImA250 | Nucleic Acid Metabolism | Nuclear ubiquitous casein and cyclin-dependent kinases substrate (P1) | EE745156 |
| T003C11 | Nucleic Acid Metabolism | Polyadenylate-binding protein 2 | EE744254 |
| T001H08 | Nucleic Acid Metabolism | Polyadenylate-binding protein 2 | EE744897 |
| TImA487 | Nucleic Acid Metabolism | Polypyrimidine tract-binding protein 1 | EE744005 |
| TImA234 | Nucleic Acid Metabolism | Possible global transcription activator SNF2L2 | EE745143 |
| T006G02 | Nucleic Acid Metabolism | Probable ATP-dependent RNA helicase | EE744527 |
| T008B01 | Nucleic Acid Metabolism | Probable RNA dependent helicase p68 | EE744622 |
| T007H01 | Nucleic Acid Metabolism | Probable RNA-dependent helicase p68 | EE744603 |
| TImA764 | Nucleic Acid Metabolism | Protein LRP16 | EE744215 |
| TImA229 | Nucleic Acid Metabolism | Putative pre-mRNA splicing factor ATP-dependent RNA helicase | EE745139 |
| T012E12 | Nucleic Acid Metabolism | Ras-related protein Rap-1A | EE744949 |
| T003B12 | Nucleic Acid Metabolism | RNA dependent helicase p68 | EE744222 |
| TImA280 | Nucleic Acid Metabolism | similar to heterogeneous nuclear ribonucleoprotein C isoform b | EE745179 |
| T001G04 | Nucleic Acid Metabolism | Similar to mSin3A-associated protein | EE744764 |
| T005H04 | Nucleic Acid Metabolism | similar to serine/arginine repetitive matrix 2 | EE744455 |
| T008E12 | Nucleic Acid Metabolism | similar to splicing factor, arginine/serine-rich 2, interacting protein | EE744668 |
| T005E03 | Nucleic Acid Metabolism | Similar to splicing factor, arginine/serine-rich isoform 1 | EE744419 |
| TImA723 | Nucleic Acid Metabolism | Small EDRK-rich factor 2 | EE744180 |
| T003B03 | Nucleic Acid Metabolism | Small EDRK-rich factor 2 (4F5rel) | EE744122 |
| T008F03 | Nucleic Acid Metabolism | small nucleolar ribonucleoprotein component | EE744671 |
| TImA702 | Nucleic Acid Metabolism | Smu-1 | EE744159 |
| TImA514 | Nucleic Acid Metabolism | TAF12 RNA polymerase | EE744028 |
| TImA268 | Nucleic Acid Metabolism | tat interactive protein homolog | EE745167 |
| TImA138 | Nucleic Acid Metabolism | Transcription elongation factor B polypeptide 2 | EE745089 |
| T003F04 | Nucleic Acid Metabolism | U1 small nuclear ribonucleoprotein A | EE744280 |
| T005F06 | Nucleic Acid Metabolism | U6 snRNA-associated Sm-like protein LSm3 | EE744433 |
| T013C11 | Nucleic Acid Metabolism | UV excision repair protein | EE745011 |
| T003E06 | Nucleic Acid Metabolism | Vaccinia-related kinase 2 | EE744273 |
| TImA425 | Nucleic Acid Metabolism | WD-repeat protein 39 | EE743951 |
| T010E06 | Nucleic Acid Metabolism | Zinc finger CCCH-type domain containing protein 7A | EE744834 |
| T007B02 | Nucleic Acid Metabolism | Zinc finger protein 323 | EE744552 |
| T006E01 | Nucleic Acid Metabolism | Zinc finger protein 347 | EE744504 |
| T012H08 | Nucleic Acid Metabolism | Zinc finger protein 38 | EE744973 |
| TImA135 | Nucleic Acid Metabolism | Zinc finger protein 501 | EE745087 |
| T008F07 | Nucleic Acid Metabolism | Zinc Finger protein 592 | EE744676 |
| TImA456 | Nucleic Acid Metabolism | Zinc finger protein 706 | EE743980 |
| TImA784 | Nucleic Acid Metabolism | Zinc finger protein BCL11B | EE744229 |
| T008G02 | Nucleic Acid Metabolism | Zinc finger protein-like 1 | EE744681 |
| T012C04 | Protein Metabolism | 16S ribosomal gene | EE744931 |
| TImA103 | Protein Metabolism | 26S protease regulatory subunit 8 | EE745066 |
| T004H02 | Protein Metabolism | 26S protease regulatory subunit S10B | EE744369 |
| TImA230 | Protein Metabolism | 28S ribosomal protein S11, mitochondrial precursor | EE745140 |
| T012F05 | Protein Metabolism | 28S rRNA gene | EE744953 |
| T010F01 | Protein Metabolism | 39S ribosomal protein L9 | EE744840 |
| T003C01 | Protein Metabolism | 40S ribosomal protein S10 | EE744233 |
| T006C11 | Protein Metabolism | 40S ribosomal protein S10 | EE744492 |
| T011E10 | Protein Metabolism | 40S ribosomal protein S10 | EE744891 |
| TImA710 | Protein Metabolism | 40S ribosomal protein S10 | EE744167 |
| T003D07 | Protein Metabolism | 40S ribosomal protein S11 | EE744263 |
| T009F08 | Protein Metabolism | 40S ribosomal protein S11 | EE744758 |
| T003B08 | Protein Metabolism | 40S ribosomal protein S12 | EE744177 |
| TImA792 | Protein Metabolism | 40S ribosomal protein S12 | EE744237 |
| T004B11 | Protein Metabolism | 40S ribosomal protein S13 | EE744322 |
| T007A11 | Protein Metabolism | 40S ribosomal protein S13 | EE744550 |
| TImA150 | Protein Metabolism | 40S ribosomal protein S13 | EE745099 |
| T004E06 | Protein Metabolism | 40S ribosomal protein S14 | EE744344 |
| T009H06 | Protein Metabolism | 40S ribosomal protein S14 | EE744781 |
| T012G10 | Protein Metabolism | 40S ribosomal protein S14 | EE744966 |
| TImA156 | Protein Metabolism | 40S ribosomal protein S14 | EE745102 |
| T013A04 | Protein Metabolism | 40S ribosomal protein S15 (RIG protein) | EE744981 |
| TImA388 | Protein Metabolism | 40S ribosomal protein S15 (RIG protein) | EE743918 |
| T005A12 | Protein Metabolism | 40S ribosomal protein S15a | EE744383 |
| T008B05 | Protein Metabolism | 40S ribosomal protein S16 | EE744626 |
| T009E07 | Protein Metabolism | 40S ribosomal protein S16 | EE744746 |
| T013G11 | Protein Metabolism | 40S ribosomal protein S16 | EE745053 |
| T003E12 | Protein Metabolism | 40S ribosomal protein S17 | EE744276 |
| T005B03 | Protein Metabolism | 40S ribosomal protein S17 | EE744385 |
| T005G12 | Protein Metabolism | 40S ribosomal protein S17 | EE744450 |
| T006E06 | Protein Metabolism | 40S ribosomal protein S17 | EE744509 |
| T012G09 | Protein Metabolism | 40S ribosomal protein S17 | EE744965 |
| TImA534 | Protein Metabolism | 40S ribosomal protein S17 | EE744047 |
| T007F12 | Protein Metabolism | 40S ribosomal protein S18 | EE744593 |
| T008B06 | Protein Metabolism | 40S ribosomal protein S18 | EE744627 |
| TImA368 | Protein Metabolism | 40S ribosomal protein S18 | EE743904 |
| T004F10 | Protein Metabolism | 40S ribosomal protein S19 | EE744356 |
| T009H05 | Protein Metabolism | 40S ribosomal protein S19 | EE744780 |
| T008D11 | Protein Metabolism | 40S ribosomal protein S19 | EE744657 |
| TImA204 | Protein Metabolism | 40S ribosomal protein S19 | EE745128 |
| TImA294 | Protein Metabolism | 40S ribosomal protein S19 | EE745193 |
| TImA386 | Protein Metabolism | 40S ribosomal protein S19 | EE743916 |
| TImA681 | Protein Metabolism | 40S ribosomal protein S19 | EE744145 |
| T001A02 | Protein Metabolism | 40S ribosomal protein S2 | EE744652 |
| T006B11 | Protein Metabolism | 40S ribosomal protein S2 | EE744481 |
| TImA37 | Protein Metabolism | 40S ribosomal protein S2 | EE743906 |
| T007A08 | Protein Metabolism | 40S ribosomal protein S20 | EE744548 |
| TImA191 | Protein Metabolism | 40S ribosomal protein S20 | EE745120 |
| TImA735 | Protein Metabolism | 40S ribosomal protein S23 | EE744189 |
| T013B02 | Protein Metabolism | 40S ribosomal protein S24 | EE744991 |
| TImA159 | Protein Metabolism | 40S ribosomal protein S24 | EE745105 |
| T004E08 | Protein Metabolism | 40S ribosomal protein S25 | EE744345 |
| T008D01 | Protein Metabolism | 40S ribosomal protein S25 | EE744646 |
| T012A09 | Protein Metabolism | 40S ribosomal protein S25 | EE744915 |
| T002G02 | Protein Metabolism | 40S ribosomal protein S26 | EE743900 |
| T001F10 | Protein Metabolism | 40S ribosomal protein S27a | EE744697 |
| T011G09 | Protein Metabolism | 40S ribosomal protein S27a | EE744900 |
| TImA765 | Protein Metabolism | 40S ribosomal protein S29 | EE744216 |
| TImA321 | Protein Metabolism | 40S ribosomal protein S29 | EE745205 |
| T011A04 | Protein Metabolism | 40S ribosomal protein S3 | EE744868 |
| T012C11 | Protein Metabolism | 40S ribosomal protein S3 | EE744935 |
| T013H05 | Protein Metabolism | 40S ribosomal protein S3 | EE745059 |
| TImA548 | Protein Metabolism | 40S ribosomal protein S3 | EE744061 |
| TImA670 | Protein Metabolism | 40S ribosomal protein S3 | EE744135 |
| T008E04 | Protein Metabolism | 40S ribosomal protein S30 | EE744660 |
| T001D06 | Protein Metabolism | 40S ribosomal protein S3A | EE744453 |
| T006E07 | Protein Metabolism | 40S ribosomal protein S3a | EE744510 |
| T006H02 | Protein Metabolism | 40S ribosomal protein S3A | EE744535 |
| T012H10 | Protein Metabolism | 40S ribosomal protein S3a | EE744976 |
| T003H05 | Protein Metabolism | 40S ribosomal protein S4 | EE744302 |
| T008C07 | Protein Metabolism | 40S ribosomal protein S4 | EE744639 |
| TImA758 | Protein Metabolism | 40S ribosomal protein S4 | EE744209 |
| T007H11 | Protein Metabolism | 40S ribosomal protein S6 | EE744610 |
| T008D06 | Protein Metabolism | 40S ribosomal protein S6 | EE744651 |
| T011H04 | Protein Metabolism | 40S ribosomal protein S6 | EE744903 |
| TImA676 | Protein Metabolism | 40S ribosomal protein S6 | EE744140 |
| TImA678 | Protein Metabolism | 40S ribosomal protein S6 | EE744141 |
| T006H03 | Protein Metabolism | 40S ribosomal protein S7 | EE744536 |
| T009E11 | Protein Metabolism | 40S ribosomal protein S7 | EE744750 |
| T010E07 | Protein Metabolism | 40S ribosomal protein S7 | EE744835 |
| T008E09 | Protein Metabolism | 40S ribosomal protein S7 | EE744665 |
| TImA95 | Protein Metabolism | 40S ribosomal protein S8 | EE744242 |
| T003H02 | Protein Metabolism | 40S ribosomal protein S9 | EE744298 |
| T001B12 | Protein Metabolism | 40S ribosomal protein SA | EE744321 |
| TImA736 | Protein Metabolism | 40S ribosomal protein SA | EE744190 |
| T009B06 | Protein Metabolism | 40S ribosomal protein SA | EE744713 |
| T010H01 | Protein Metabolism | 40S ribosomal protein SA | EE744860 |
| T011A12 | Protein Metabolism | 40S ribosomal protein SA | EE744872 |
| T012D08 | Protein Metabolism | 40S ribosomal protein SA | EE744940 |
| T008G11 | Protein Metabolism | 40S ribosomal protein SA | EE744690 |
| TImA728 | Protein Metabolism | 40S ribosomal protein SA (p40) (C10 protein) | EE744183 |
| T005H10 | Protein Metabolism | 60S acidic ribosomal protein P0 | EE744459 |
| T009G11 | Protein Metabolism | 60S acidic ribosomal protein P0 | EE744774 |
| T003D03 | Protein Metabolism | 60S acidic ribosomal protein P1 | EE744259 |
| T008E02 | Protein Metabolism | 60S acidic ribosomal protein P1 | EE744659 |
| TImA563 | Protein Metabolism | 60S acidic ribosomal protein P1 | EE744075 |
| T011G08 | Protein Metabolism | 60S acidic ribosomal protein P2 | EE744899 |
| T001H05 | Protein Metabolism | 60S ribosomal protein L10 | EE744875 |
| TImA752 | Protein Metabolism | 60S ribosomal protein L10 | EE744205 |
| T010G12 | Protein Metabolism | 60S ribosomal protein L10 | EE744859 |
| T011C08 | Protein Metabolism | 60S ribosomal protein L10 | EE744881 |
| TImA445 | Protein Metabolism | 60S ribosomal protein L10 | EE743972 |
| TImA53 | Protein Metabolism | 60S ribosomal protein L10 | EE744041 |
| T013A05 | Protein Metabolism | 60S ribosomal protein L10a (CSA-19) | EE744982 |
| T009F11 | Protein Metabolism | 60S ribosomal protein L11 | EE744761 |
| T010F04 | Protein Metabolism | 60S ribosomal protein L11 | EE744843 |
| T013F02 | Protein Metabolism | 60S ribosomal protein L11 | EE745036 |
| TImA197 | Protein Metabolism | 60S ribosomal protein L11 | EE745124 |
| TImA236 | Protein Metabolism | 60S ribosomal protein L11 | EE745145 |
| TImA392 | Protein Metabolism | 60S ribosomal protein L11 | EE743921 |
| TImA604 | Protein Metabolism | 60S ribosomal protein L11 | EE744100 |
| TImA718 | Protein Metabolism | 60S ribosomal protein L11 | EE744175 |
| TImA14 | Protein Metabolism | 60S ribosomal protein L11 | EE745090 |
| T001F12 | Protein Metabolism | 60S ribosomal protein L12 | EE744719 |
| TImA631 | Protein Metabolism | 60S ribosomal protein L12 | EE744114 |
| T006F05 | Protein Metabolism | 60S ribosomal protein L13 | EE744518 |
| TImA786 | Protein Metabolism | 60S ribosomal protein L13 | EE744231 |
| T012B07 | Protein Metabolism | 60S ribosomal protein L13 | EE744921 |
| T013D07 | Protein Metabolism | 60S ribosomal protein L13 (A52) | EE745018 |
| TImA287 | Protein Metabolism | 60S ribosomal protein L13 (A52) | EE745186 |
| TImA524 | Protein Metabolism | 60S ribosomal protein L13 (A52) | EE744035 |
| TImA547 | Protein Metabolism | 60S ribosomal protein L13 (A52) | EE744060 |
| TImA708 | Protein Metabolism | 60S ribosomal protein L13 (A52) | EE744163 |
| T006F07 | Protein Metabolism | 60S ribosomal protein L13a | EE744521 |
| TImA747 | Protein Metabolism | 60S ribosomal protein L13a | EE744201 |
| TImA55 | Protein Metabolism | 60S ribosomal protein L13a | EE744063 |
| T003B01 | Protein Metabolism | 60S ribosomal protein L14 | EE744099 |
| T003F10 | Protein Metabolism | 60S ribosomal protein L14 | EE744285 |
| TImA238 | Protein Metabolism | 60S ribosomal protein L14 (CAG-ISL 7) | EE745147 |
| TImA369 | Protein Metabolism | 60S ribosomal protein L14 (CAG-ISL 7) | EE743905 |
| T004E11 | Protein Metabolism | 60S ribosomal protein L15 | EE744348 |
| T005D04 | Protein Metabolism | 60S ribosomal protein L15 | EE744407 |
| TImA237 | Protein Metabolism | 60S ribosomal protein L15 | EE745146 |
| TImA337 | Protein Metabolism | 60S ribosomal protein L15 | EE743892 |
| TImA410 | Protein Metabolism | 60S ribosomal protein L15 | EE743940 |
| TImA626 | Protein Metabolism | 60S ribosomal protein L15 | EE744112 |
| TImA186 | Protein Metabolism | 60S ribosomal protein L15 (L10) | EE745116 |
| T004H03 | Protein Metabolism | 60S ribosomal protein L17 | EE744370 |
| TImA203 | Protein Metabolism | 60S ribosomal protein L17 | EE745127 |
| TImA395 | Protein Metabolism | 60S ribosomal protein L17 | EE743925 |
| T001G07 | Protein Metabolism | 60S ribosomal protein L18 | EE744797 |
| T005C12 | Protein Metabolism | 60S ribosomal protein L18 | EE744405 |
| T009C06 | Protein Metabolism | 60S ribosomal protein L18 | EE744722 |
| T013E02 | Protein Metabolism | 60S ribosomal protein L18 | EE745025 |
| T013F01 | Protein Metabolism | 60S ribosomal protein L18 | EE745035 |
| T004F08 | Protein Metabolism | 60S ribosomal protein L18a | EE744355 |
| T001D08 | Protein Metabolism | 60S ribosomal protein L19 | EE744475 |
| T009E02 | Protein Metabolism | 60S ribosomal protein L19 | EE744740 |
| T008D10 | Protein Metabolism | 60S ribosomal protein L19 | EE744656 |
| TImA517 | Protein Metabolism | 60S ribosomal protein L19 | EE744030 |
| TImA675 | Protein Metabolism | 60S ribosomal protein L19 | EE744139 |
| T013C07 | Protein Metabolism | 60S ribosomal protein L21 | EE745007 |
| T013D10 | Protein Metabolism | 60S ribosomal protein L21 | EE745022 |
| T002G08 | Protein Metabolism | 60S ribosomal protein L22 | EE743944 |
| T004F03 | Protein Metabolism | 60S ribosomal protein L22 | EE744350 |
| T006C12 | Protein Metabolism | 60S ribosomal protein L22 | EE744493 |
| T004B06 | Protein Metabolism | 60S ribosomal protein L23 | EE744320 |
| T007B01 | Protein Metabolism | 60S ribosomal protein L23 | EE744551 |
| T009C04 | Protein Metabolism | 60S ribosomal protein L23 | EE744720 |
| T010H12 | Protein Metabolism | 60S ribosomal protein L23 | EE744866 |
| T013D06 | Protein Metabolism | 60S ribosomal protein L23 | EE745017 |
| TImA158 | Protein Metabolism | 60S ribosomal protein L23 | EE745104 |
| TImA370 | Protein Metabolism | 60S ribosomal protein L23 | EE743907 |
| T008G03 | Protein Metabolism | 60S ribosomal protein L23a | EE744682 |
| T001E12 | Protein Metabolism | 60S ribosomal protein L26 | EE744608 |
| T003B04 | Protein Metabolism | 60S ribosomal protein L26 | EE744133 |
| T004B01 | Protein Metabolism | 60S ribosomal protein L26 | EE744317 |
| T004D12 | Protein Metabolism | 60S ribosomal protein L26 | EE744339 |
| T005D12 | Protein Metabolism | 60S ribosomal protein L26 | EE744416 |
| T005G11 | Protein Metabolism | 60S ribosomal protein L26 | EE744449 |
| T006C01 | Protein Metabolism | 60S ribosomal protein L26 | EE744482 |
| T012C10 | Protein Metabolism | 60S ribosomal protein L26 | EE744934 |
| T013F08 | Protein Metabolism | 60S ribosomal protein L26 | EE745040 |
| TImA115 | Protein Metabolism | 60S ribosomal protein L26 | EE745073 |
| TImA566 | Protein Metabolism | 60S ribosomal protein L26 | EE744078 |
| TImA27 | Protein Metabolism | 60S ribosomal protein L26 | EE745169 |
| T006F06 | Protein Metabolism | 60S ribosomal protein L27 | EE744520 |
| T003D12 | Protein Metabolism | 60S ribosomal protein L27a | EE744268 |
| TImB301 | Protein Metabolism | 60S ribosomal protein L27a | EE744247 |
| T004G05 | Protein Metabolism | 60S ribosomal protein L28 | EE744362 |
| T006B09 | Protein Metabolism | 60S ribosomal protein L28 | EE744479 |
| T008B12 | Protein Metabolism | 60S ribosomal protein L28 | EE744633 |
| T008C12 | Protein Metabolism | 60S ribosomal protein L28 | EE744645 |
| TImA768 | Protein Metabolism | 60S ribosomal protein L28 | EE744217 |
| T012B08 | Protein Metabolism | 60S ribosomal protein L28 | EE744922 |
| T013E08 | Protein Metabolism | 60S ribosomal protein L28 | EE745031 |
| T012B12 | Protein Metabolism | 60S ribosomal protein L3 (L4) | EE744926 |
| T001H03 | Protein Metabolism | 60S ribosomal protein L30 | EE744852 |
| T013E11 | Protein Metabolism | 60S ribosomal protein L30 | EE745034 |
| T004D06 | Protein Metabolism | 60S ribosomal protein L31 | EE744335 |
| T004E09 | Protein Metabolism | 60S ribosomal protein L31 | EE744346 |
| T010A08 | Protein Metabolism | 60S ribosomal protein L31 | EE744792 |
| T007D05 | Protein Metabolism | 60S ribosomal protein L34 | EE744573 |
| T006H07 | Protein Metabolism | 60S ribosomal protein L35 | EE744540 |
| T007D10 | Protein Metabolism | 60S ribosomal protein L35 | EE744576 |
| TImA414 | Protein Metabolism | 60S ribosomal protein L35 | EE743943 |
| T007E05 | Protein Metabolism | 60S ribosomal protein L36 | EE744580 |
| T008F11 | Protein Metabolism | 60S ribosomal protein L36 | EE744678 |
| TImA673 | Protein Metabolism | 60S ribosomal protein L36a | EE744137 |
| T004D09 | Protein Metabolism | 60S ribosomal protein L36a-like | EE744337 |
| T003B07 | Protein Metabolism | 60S ribosomal protein L37 | EE744166 |
| T010D01 | Protein Metabolism | 60S ribosomal protein L37a | EE744820 |
| T008H08 | Protein Metabolism | 60S ribosomal protein L37a | EE744698 |
| T003B11 | Protein Metabolism | 60S ribosomal protein L38 | EE744210 |
| TImA270 | Protein Metabolism | 60S ribosomal protein L39 | EE745170 |
| T004A08 | Protein Metabolism | 60S ribosomal protein L4 | EE744313 |
| T013F09 | Protein Metabolism | 60S ribosomal protein L4 (L1) | EE745042 |
| TImA242 | Protein Metabolism | 60S ribosomal protein L40 (CEP52) | EE745149 |
| T001A09 | Protein Metabolism | 60S ribosomal protein L5 | EE743888 |
| T003C04 | Protein Metabolism | 60S ribosomal protein L5 | EE744248 |
| T007G11 | Protein Metabolism | 60S ribosomal protein L5 | EE744601 |
| TImA424 | Protein Metabolism | 60S ribosomal protein L5 | EE743950 |
| T005E08 | Protein Metabolism | 60S ribosomal protein L6 | EE744423 |
| T007F05 | Protein Metabolism | 60S ribosomal protein L6 | EE744588 |
| T009D03 | Protein Metabolism | 60S ribosomal protein L6 | EE744731 |
| T003H12 | Protein Metabolism | 60S ribosomal protein L7 | EE744307 |
| T004F06 | Protein Metabolism | 60S ribosomal protein L7 | EE744353 |
| TImA69 | Protein Metabolism | 60S ribosomal protein L7 | EE744148 |
| T004G12 | Protein Metabolism | 60S ribosomal protein L8 | EE744368 |
| TImA674 | Protein Metabolism | 60S ribosomal protein L8 | EE744138 |
| T005H08 | Protein Metabolism | 60S ribosomal protein L9 | EE744457 |
| T007D01 | Protein Metabolism | 60S ribosomal protein L9 | EE744570 |
| T012A02 | Protein Metabolism | 60S ribosomal protein L9 | EE744912 |
| TImA285 | Protein Metabolism | 60S ribosomal protein L9 | EE745184 |
| T004C02 | Protein Metabolism | Alpha-1,6-mannosyl-glycoprotein 2-beta-N-acetylglucosaminyltransferase | EE744325 |
| TImA512 | Protein Metabolism | Aminoacylase-1 | EE744026 |
| TImA415 | Protein Metabolism | cAMP-dependent protein kinase type I-alpha regulatory subunit | EE743945 |
| T005D11 | Protein Metabolism | Cathepsin J | EE744415 |
| T002A07 | Protein Metabolism | Cathepsin L precursor | EE744974 |
| TImA647 | Protein Metabolism | cullin 1 | EE744124 |
| TImA6 | Protein Metabolism | cyclophilin A | EE744096 |
| T004G03 | Protein Metabolism | DnaJ homolog subfamily C member 9 | EE744360 |
| T007C07 | Protein Metabolism | Dual specificity testis-specific protein kinase 2 | EE744565 |
| T004B02 | Protein Metabolism | Elongation factor 1-alpha | EE744318 |
| T007D09 | Protein Metabolism | Elongation factor 1-alpha | EE744574 |
| T008C04 | Protein Metabolism | Elongation factor 1-alpha | EE744637 |
| T013D03 | Protein Metabolism | Elongation factor 1-alpha 1 | EE745014 |
| T013E01 | Protein Metabolism | Elongation factor 1-alpha 1 | EE745024 |
| T008H04 | Protein Metabolism | Elongation factor 1-alpha, somatic form | EE744693 |
| T001C03 | Protein Metabolism | Elongation factor 1-beta | EE744343 |
| T007C10 | Protein Metabolism | Elongation factor 1-delta | EE744568 |
| TImA397 | Protein Metabolism | Elongation factor 1-delta | EE743926 |
| TImA639 | Protein Metabolism | elongation factor-1 alpha | EE744118 |
| T012H09 | Protein Metabolism | Eukaryotic initiation factor 4A-I | EE744975 |
| T012H11 | Protein Metabolism | Eukaryotic initiation factor 4A-I | EE744977 |
| TImA304 | Protein Metabolism | eukaryotic translation elongation factor 1 alpha 1 | EE745199 |
| TImA194 | Protein Metabolism | Eukaryotic translation initiation factor 3 subunit 12 | EE745123 |
| T013E05 | Protein Metabolism | Eukaryotic translation initiation factor 3 subunit 2 | EE745027 |
| TImA703 | Protein Metabolism | Eukaryotic translation initiation factor 3 subunit 3 | EE744160 |
| T011D06 | Protein Metabolism | FK506-binding protein 1A | EE744885 |
| T002G04 | Protein Metabolism | Glycyl-tRNA synthetase | EE743922 |
| TImA435 | Protein Metabolism | Heat shock protein 75 kDa, mitochondrial precursor | EE743962 |
| T001F04 | Protein Metabolism | Heat Shock protein HSP 90-beta | EE744653 |
| T005G08 | Protein Metabolism | Heat shock protein HSP 90-beta | EE744447 |
| T006B10 | Protein Metabolism | Heat shock protein HSP 90-beta | EE744480 |
| T009D05 | Protein Metabolism | Leucine carboxyl methyltransferase | EE744733 |
| T011H03 | Protein Metabolism | Ligatin | EE744902 |
| TImA538 | Protein Metabolism | Methionine-R-sulfoxide reductase B2 | EE744050 |
| T008E11 | Protein Metabolism | Microsomal signal peptidase 18 kDa subunit | EE744667 |
| TImA387 | Protein Metabolism | Microsomal signal peptidase 21 kDa subunit | EE743917 |
| T007G12 | Protein Metabolism | Neuroserpin precursor | EE744602 |
| T012D01 | Protein Metabolism | Neuroserpin precursor | EE744937 |
| TImA84 | Protein Metabolism | Neuroserpin precursor | EE744239 |
| T012F12 | Protein Metabolism | PAB-dependent poly(A)-specific ribonuclease subunit PAN2 | EE744960 |
| T003D09 | Protein Metabolism | Peptidyl-prolyl cis-trans isomerase A | EE744264 |
| TImA731 | Protein Metabolism | Peptidyl-prolyl cis-trans isomerase A | EE744186 |
| T012F11 | Protein Metabolism | Peptidyl-prolyl cis-trans isomerase A | EE744959 |
| TImA247 | Protein Metabolism | Peptidyl-prolyl cis-trans isomerase A | EE745154 |
| TImA540 | Protein Metabolism | Peptidyl-prolyl cis-trans isomerase A | EE744053 |
| T011H11 | Protein Metabolism | Peptidyl-prolyl cis-trans isomerase A | EE744910 |
| TImA692 | Protein Metabolism | Peptidyl-prolyl cis-trans isomerase A | EE744151 |
| T010B05 | Protein Metabolism | Poly (ADP-ribose) polymerase family, member 16 | EE744801 |
| TImA699 | Protein Metabolism | Porphobilinogen deaminase | EE744156 |
| T001A04 | Protein Metabolism | Prefoldin subunit 3 | EE744763 |
| T009H03 | Protein Metabolism | Prenylated Rab acceptor protein 1 | EE744778 |
| TImA210 | Protein Metabolism | Probable 60S ribosomal protein L37-A | EE745136 |
| T007H07 | Protein Metabolism | Prostate, ovary, testis expressed protein on chromosome 8 | EE744606 |
| T013A11 | Protein Metabolism | Proteasome activator complex subunit 1 | EE744988 |
| TImA155 | Protein Metabolism | Proteasome subunit alpha type 2 | EE745101 |
| T001A06 | Protein Metabolism | Proteasome subunit beta type 1 | EE744874 |
| T007B04 | Protein Metabolism | Proteasome subunit beta type 1 | EE744554 |
| TImA593 | Protein Metabolism | Protein tyrosine phosphatase type IVA protein 2 | EE744091 |
| TImA129 | Protein Metabolism | Proteosome subunit | EE745081 |
| T005B08 | Protein Metabolism | PTD016 protein | EE744390 |
| T010G09 | Protein Metabolism | ribosomal protein L40 | EE744858 |
| T013B11 | Protein Metabolism | S-phase kinase-associated protein 1A | EE745000 |
| TImA288 | Protein Metabolism | Sequestosome-1 | EE745187 |
| T005H03 | Protein Metabolism | Signal recognition particle receptor alpha subunit | EE744454 |
| TImA254 | Protein Metabolism | similar to nucleophosmin 1 | EE745159 |
| TImA672 | Protein Metabolism | sorting nexin 12 | EE744136 |
| T009C11 | Protein Metabolism | Splicing factor, arginin/serine-rich 1 | EE744727 |
| T004A05 | Protein Metabolism | Stromal cell-derived factor 2 | EE744311 |
| T004C10 | Protein Metabolism | T-complex protein 1 | EE744329 |
| T005F10 | Protein Metabolism | T-complex protein 1 | EE744437 |
| T006H05 | Protein Metabolism | T-complex protein 1 | EE744538 |
| TImA434 | Protein Metabolism | T-complex protein 1, gamma subunit | EE743961 |
| T009H07 | Protein Metabolism | Transcription elongation factor B polypeptide 2 | EE744782 |
| TImA241 | Protein Metabolism | translation elongation factor 1 alpha 1 | EE745148 |
| TImA762 | Protein Metabolism | Translationally controlled tumor protein | EE744213 |
| T005H11 | Protein Metabolism | Tripeptidyl-peptidase I precursor | EE744460 |
| T001F11 | Protein Metabolism | Ubiquitin | EE744708 |
| T002B08 | Protein Metabolism | Ubiquitin | EE745052 |
| T003F12 | Protein Metabolism | Ubiquitin | EE744286 |
| T005F05 | Protein Metabolism | Ubiquitin | EE744432 |
| T006C03 | Protein Metabolism | Ubiquitin | EE744483 |
| T006D04 | Protein Metabolism | Ubiquitin | EE744498 |
| T007B05 | Protein Metabolism | Ubiquitin | EE744555 |
| T008A05 | Protein Metabolism | Ubiquitin | EE744614 |
| T009B02 | Protein Metabolism | Ubiquitin | EE744712 |
| T009D09 | Protein Metabolism | Ubiquitin | EE744737 |
| T009D10 | Protein Metabolism | Ubiquitin | EE744738 |
| T011F07 | Protein Metabolism | Ubiquitin | EE744894 |
| T013B10 | Protein Metabolism | Ubiquitin | EE744999 |
| T008F05 | Protein Metabolism | Ubiquitin | EE744673 |
| TImA379 | Protein Metabolism | Ubiquitin | EE743909 |
| TImA269 | Protein Metabolism | ubiquitin conjugating enzyme | EE745168 |
| T009H02 | Protein Metabolism | Ubiquitin like containing PHD and RING finger domains protein 1 | EE744777 |
| TImA746 | Protein Metabolism | Ubiquitin specific peptidase 28 | EE744200 |
| T001A11 | Protein Metabolism | Ubiquitin-conjugating enzyme E2 B | EE743999 |
| T010A07 | Protein Metabolism | Ubiquitin-like 1 activating enzyme E1B | EE744791 |
| T002H02 | Protein Metabolism | Ubiquitin-like protein FUBI | EE743955 |
| T003G09 | Signal Transduction | 14-3-3 protein theta | EE744294 |
| TImA741 | Signal Transduction | ADP-ribosylation factor 1 | EE744195 |
| T002F04 | Signal Transduction | Amphiphysin-like protein | EE745174 |
| T001E10 | Signal Transduction | Angiotensinogen precursor | EE744586 |
| T005B05 | Signal Transduction | Angiotensinogen precursor | EE744388 |
| T008B08 | Signal Transduction | Angiotensinogen precursor | EE744629 |
| T009D04 | Signal Transduction | Angiotensinogen precursor | EE744732 |
| T002G03 | Signal Transduction | Calcitonin gene-related peptide-receptor component protein | EE743911 |
| T006E03 | Signal Transduction | Calgizzarin | EE744506 |
| TImA161 | Signal Transduction | cell cycle related kinase | EE745106 |
| T007D12 | Signal Transduction | Cirhin | EE744577 |
| TImA198 | Signal Transduction | COMM domain containing protein 3 | EE745125 |
| T002F07 | Signal Transduction | Cysteine-rich protein 1 | EE745196 |
| T003F03 | Signal Transduction | Dedicator of cytokinesis protein 8 | EE744279 |
| TImA109 | Signal Transduction | Diacylglycerol kinase, epsilon | EE745068 |
| T006G12 | Signal Transduction | Docking protein 2 | EE744533 |
| T005C01 | Signal Transduction | Flotillin-2 | EE744395 |
| T006B01 | Signal Transduction | Glia maturation factor gamma | EE744471 |
| TImA130 | Signal Transduction | Glia maturation factor gamma | EE745082 |
| TImA748 | Signal Transduction | Growth factor receptor bound protein 2 | EE744202 |
| T005H12 | Signal Transduction | GTP binding protein | EE744461 |
| T010D02 | Signal Transduction | GTP-binding nuclear protein Ran | EE744821 |
| T004E10 | Signal Transduction | Guanine nucleotide-binding protein beta subunit 2-like 1 | EE744347 |
| T006C09 | Signal Transduction | Guanine nucleotide-binding protein beta subunit 2-like 1 | EE744490 |
| T006H06 | Signal Transduction | Guanine nucleotide-binding protein beta subunit 2-like 1 | EE744539 |
| T010C12 | Signal Transduction | Guanine nucleotide-binding protein beta subunit 2-like 1 | EE744818 |
| TImA442 | Signal Transduction | Guanine nucleotide-binding protein beta subunit 2-like 1 | EE743969 |
| TImA29 | Signal Transduction | Guanine nucleotide-binding protein beta subunit 2-like 1 | EE745189 |
| T013F12 | Signal Transduction | Guanylate cyclase soluble, alpha-3 chain | EE745045 |
| T006H01 | Signal Transduction | Importin-4 | EE744534 |
| T011H09 | Signal Transduction | Integrin-linked protein kinase 1 | EE744909 |
| TImA353 | Signal Transduction | Metabotropic glutamate receptor 6 precursor | EE743897 |
| T011G01 | Signal Transduction | Mitogen-activated protein kinase kinase 1 interacting protein 1 | EE744895 |
| T001A01 | Signal Transduction | Mitogen-activated protein kinase kinase kinase kinase 2 | EE744541 |
| T003B02 | Signal Transduction | Mitogen-activated protein kinase kinase kinase kinase 2 | EE744111 |
| T008A02 | Signal Transduction | OPN1MW | EE744612 |
| T010B11 | Signal Transduction | Parathyroid hormone precursor | EE744807 |
| T010E04 | Signal Transduction | Phosphatidylinositol-4-phosphate 5-kinase type II alpha | EE744832 |
| TImA22 | Signal Transduction | plexin 1 | EE745137 |
| T003F08 | Signal Transduction | PP2A, subunit A, R1-alpha isoform | EE744283 |
| TImA474 | Signal Transduction | proline rich nuclear receptor coactivator 1 | EE743992 |
| TImA472 | Signal Transduction | Protein phosphatase 4 subunit 2 | EE743991 |
| TImA452 | Signal Transduction | Protein tyrosine phosphatase type IVA protein 2 | EE743976 |
| T006E08 | Signal Transduction | Rap guanine nucleotide exchange factor | EE744511 |
| T011D10 | Signal Transduction | RAS guanyl releasing protein 3 | EE744887 |
| T008H11 | Signal Transduction | Ras-related C3 botulinum toxin substrate 2 | EE744701 |
| T006D03 | Signal Transduction | Rho GDP-dissociation inhibitor 2 | EE744496 |
| T009F05 | Signal Transduction | Rho-GTPase activating protein 9 | EE744755 |
| T005A04 | Signal Transduction | Rho-GTPase-activating protein 25 | EE744378 |
| T010E09 | Signal Transduction | RhoA GTPase effector | EE744837 |
| TImA192 | Signal Transduction | RhoA GTPase effector | EE745121 |
| TImA502 | Signal Transduction | RTN1 | EE744017 |
| TImA565 | Signal Transduction | S100 calcium-binding protein A4 | EE744076 |
| T004H09 | Signal Transduction | Serine/threonine protein phosphatase 4 catalytic subunit | EE744372 |
| TImA724 | Signal Transduction | Serine/threonine-protein kinase 12 | EE744181 |
| T001D01 | Signal Transduction | SH2 domain protein 2A | EE744409 |
| T005C02 | Signal Transduction | similar to anaphase promoting complex subunit 13 | EE744396 |
| T007D02 | Signal Transduction | Similar to proliferator-activated receptor binding protein | EE744571 |
| T008A06 | Signal Transduction | SPARC precursor | EE744615 |
| T003D04 | Signal Transduction | Syntaxin binding protein 2 | EE744260 |
| T010E05 | Signal Transduction | thyroid hormone receptor interactor 8 | EE744833 |
| T013B06 | Signal Transduction | Twinfilin-1 | EE744994 |
| T005B09 | Transcription | CCR4-NOT transcription complex subunit 8 | EE744391 |
| T009G03 | Transcription | DNA directed RNA polymerase II | EE744766 |
| TImA116 | Transcription | DNA-binding protein A | EE745075 |
| T009D06 | Transcription | DNA-directed RNA polymerase II | EE744734 |
| T009F01 | Transcription | G19 protein homolog | EE744751 |
| TImA760 | Transcription | Lamina-associated polypeptide | EE744212 |
| T006G05 | Transcription | MAD homolog 4 | EE744529 |
| T011B12 | Transcription | Myeloid/lymphoid or mixed-lineage leukemia protein 4 | EE744878 |
| T011B01 | Transcription | Nuclease sensitive element binding protein 1 | EE744873 |
| T002F02 | Transcription | Paf1/RNA polymerase II complex component | EE745152 |
| T004C04 | Transcription | PC4 and SFRS1 interacting protein | EE744327 |
| TImA573 | Transcription | PHD finger protein 3 | EE744082 |
| T002A06 | Transcription | RING finger protein 14 | EE744963 |
| T013C06 | Transcription | runt related transcription factor | EE745006 |
| TImA358 | Transcription | scaffold attachment factor B | EE743901 |
| TImA443 | Transcription | SERTA domain-containing protein 2 | EE743970 |
| T005F03 | Transcription | similar to coiled-coil transcriptional coactivator, transcript variant 1 | EE744429 |
| T009D01 | Transcription | Single-stranded DNA-binding protein 2 | EE744728 |
| T013C02 | Transcription | Staphylococcal nuclease domain containing protein 1 | EE745003 |
| T005B04 | Transcription | Transcription elongation factor A protein 1 | EE744386 |
| T005G06 | Transcription | XPA-binding protein 2 | EE744445 |
| TImA559 | Transport | ADP/ATP translocase 3 | EE744072 |
| T008D03 | Transport | ATPase, Class I, type 8B, member 2 | EE744648 |
| T013A02 | Transport | Cop-coated vesicle membrane protein p24 precursor | EE744979 |
| T008C09 | Transport | Ferritin heavy chain | EE744642 |
| T008F06 | Transport | Ferritin heavy chain | EE744674 |
| TImA664 | Transport | Ferritin heavy chain | EE744131 |
| TImA527 | Transport | Ferritin heavy chain | EE744038 |
| T006C10 | Transport | Ferritin light chain | EE744491 |
| TImA141 | Transport | Ferritin light chain | EE745092 |
| T006H04 | Transport | Ferritin light chain 1 | EE744537 |
| T001E11 | Transport | Hemoglobin alpha subunit | EE744597 |
| T008B03 | Transport | Hemoglobin alpha subunit | EE744624 |
| T002H03 | Transport | Hemoglobin beta subunit | EE743966 |
| T010D10 | Transport | Hemoglobin beta subunit | EE744829 |
| T010H09 | Transport | Hemoglobin beta subunit | EE744864 |
| T011D12 | Transport | Hemoglobin beta subunit | EE744889 |
| T013G12 | Transport | Hemoglobin beta subunit | EE745054 |
| T013H12 | Transport | Hemoglobin beta subunit | EE745065 |
| TImA516 | Transport | Hemoglobin beta subunit | EE744029 |
| TImA475 | Transport | Hemoglobin beta subunit | EE743993 |
| T006A06 | Transport | Hemoglobin beta subunit | EE744465 |
| T001G11 | Transport | Hemoglobin theta chain | EE744830 |
| T012E11 | Transport | Matrix Gla-protein precursor | EE744948 |
| TImA177 | Transport | Mitochondrial carrier homolog 2 | EE745112 |
| T003E04 | Transport | NTF2-related export protein 2 | EE744271 |
| T011G06 | Transport | Nuclear pore complex protein Nup160 | EE744898 |
| TImA98 | Transport | Nucleoporin 50 kDa, Nup50 | EE744243 |
| TImA346 | Transport | Protein transport protein Sec61 beta subunit | EE743894 |
| TImA753 | Transport | Sec1 family domain containing protein 1 | EE744206 |
| T005C04 | Transport | Serologically defined breast cancer antigen NY-BR-84 | EE744397 |
| TImA402 | Transport | similar to Exocyst complex component Sec6 | EE743931 |
| T009A02 | Transport | Spinster | EE744703 |
| T003G11 | Transport | StAR-related lipid transfer protein 7 | EE744296 |
| T011C01 | Transport | Synaptosomal-associated protein 23 | EE744879 |
| T010A04 | Transport | Thioredoxin-like protein 2 | EE744789 |
| T006E02 | Transport | Translocon-associated protein beta subunit precursor | EE744505 |
| T008E05 | Transport | Translocon-associated protein, delta subunit precursor | EE744661 |
| T003A12 | Transport | Transport protein particle 20 kDa subunit | EE744088 |
| TImA618 | Transport | Vacuolar protein sorting-associated protein 45 | EE744107 |
| T005H01 | Transport | Vesicle transport through interaction with t-SNAREs homolog 1B | EE744451 |
| TImA542 | Transport | Zinc transporter SLC39A6 precursor | EE744056 |
