# Supplementary material for: Analysis of a set of Australian northern brown bandicoot expressed sequence tags with comparison to the genome sequence of the South American grey short tailed opossum
Source: BMC Genomics. 2007 Feb 13;8:50. doi: 10.1186/1471-2164-8-50 (PMC1802078; doi:10.1186/1471-2164-8-50)
Supplement: Additional file 2 — Summary of integrated High Quality Non Coding (iHQNC) and inconsistent HQNC ESTs. Table of unmatched bandicoot ESTs that aligned to the opossum or human genomes or both. ESTs that aligned to the opossum or human genome, or both and were more than 5 kb from an annotated gene were labeled iHQNC ESTs. Those ESTs that aligned to both the human and opossum genomes, but were more than 5 kb from an annotated gene in only one species were labeled inconsistent HQNC ESTs. [file 1471-2164-8-50-S2.doc]

**Additional file 2.** Summary of integrated High Quality Non Coding (HQNC) and inconsistent HQNC ESTs

| **EST** | **Accession** | **Class** | **Aligns with human** | **Human HQNC** | **Aligns with opossum** | **Opossum HQNC** |
| --- | --- | --- | --- | --- | --- | --- |
| TImA449 | EE743974 | iHQNC | FALSE | FALSE | TRUE | TRUE |
| TImA688 | EE744146 | iHQNC | FALSE | FALSE | TRUE | TRUE |
| T003E03 | EE744270 | iHQNC | FALSE | FALSE | TRUE | TRUE |
| T008C11 | EE744644 | iHQNC | FALSE | FALSE | TRUE | TRUE |
| TImA497 | EE744012 | iHQNC | FALSE | FALSE | TRUE | TRUE |
| T001F03 | EE744641 | iHQNC | FALSE | FALSE | TRUE | TRUE |
| TImA653 | EE744125 | iHQNC | FALSE | FALSE | TRUE | TRUE |
| TImA657 | EE744128 | iHQNC | FALSE | FALSE | TRUE | TRUE |
| TImA576 | EE744083 | iHQNC | FALSE | FALSE | TRUE | TRUE |
| TImA578 | EE744085 | iHQNC | FALSE | FALSE | TRUE | TRUE |
| T004E04 | EE744341 | iHQNC | FALSE | FALSE | TRUE | TRUE |
| TImA399 | EE743928 | iHQNC | FALSE | FALSE | TRUE | TRUE |
| T001E04 | EE744553 | iHQNC | FALSE | FALSE | TRUE | TRUE |
| T013F10 | EE745043 | iHQNC | FALSE | FALSE | TRUE | TRUE |
| T007F01 | EE744587 | iHQNC | FALSE | FALSE | TRUE | TRUE |
| T006D09 | EE744501 | iHQNC | FALSE | FALSE | TRUE | TRUE |
| TImA781 | EE744227 | iHQNC | FALSE | FALSE | TRUE | TRUE |
| T005A09 | EE744382 | iHQNC | FALSE | FALSE | TRUE | TRUE |
| T010F05 | EE744844 | iHQNC | FALSE | FALSE | TRUE | TRUE |
| T003A08 | EE744055 | iHQNC | FALSE | FALSE | TRUE | TRUE |
| TImA303 | EE745198 | iHQNC | FALSE | FALSE | TRUE | TRUE |
| T005B11 | EE744393 | iHQNC | FALSE | FALSE | TRUE | TRUE |
| T005B10 | EE744392 | iHQNC | FALSE | FALSE | TRUE | TRUE |
| TImA422 | EE743948 | iHQNC | FALSE | FALSE | TRUE | TRUE |
| TImA428 | EE743954 | iHQNC | FALSE | FALSE | TRUE | TRUE |
| T004G11 | EE744367 | iHQNC | FALSE | FALSE | TRUE | TRUE |
| Contig141 | a | iHQNC | FALSE | FALSE | TRUE | TRUE |
| T009C07 | EE744723 | iHQNC | FALSE | FALSE | TRUE | TRUE |
| T008C01 | EE744634 | iHQNC | FALSE | FALSE | TRUE | TRUE |
| TImA169 | EE745111 | iHQNC | FALSE | FALSE | TRUE | TRUE |
| T003G08 | EE744293 | iHQNC | FALSE | FALSE | TRUE | TRUE |
| T003G05 | EE744291 | iHQNC | FALSE | FALSE | TRUE | TRUE |
| T003G06 | EE744292 | iHQNC | FALSE | FALSE | TRUE | TRUE |
| T003G03 | EE744289 | iHQNC | FALSE | FALSE | TRUE | TRUE |
| TImA266 | EE745165 | iHQNC | FALSE | FALSE | TRUE | TRUE |
| T010G07 | EE744856 | iHQNC | FALSE | FALSE | TRUE | TRUE |
| TImA701 | EE744158 | iHQNC | FALSE | FALSE | TRUE | TRUE |
| TImA641 | EE744120 | iHQNC | FALSE | FALSE | TRUE | TRUE |
| TImA645 | EE744121 | iHQNC | FALSE | FALSE | TRUE | TRUE |
| TImA749 | EE744203 | iHQNC | FALSE | FALSE | TRUE | TRUE |
| TImA461 | EE743984 | iHQNC | FALSE | FALSE | TRUE | TRUE |
| T007E10 | EE744585 | iHQNC | FALSE | FALSE | TRUE | TRUE |
| T006C06 | EE744487 | iHQNC | FALSE | FALSE | TRUE | TRUE |
| T003C10 | EE744253 | iHQNC | FALSE | FALSE | TRUE | TRUE |
| T008G08 | EE744688 | iHQNC | FALSE | FALSE | TRUE | TRUE |
| Contig10 | a | iHQNC | FALSE | FALSE | TRUE | TRUE |
| T013G04 | EE745047 | iHQNC | FALSE | FALSE | TRUE | TRUE |
| T009G07 | EE744770 | iHQNC | FALSE | FALSE | TRUE | TRUE |
| T013B07 | EE744995 | iHQNC | FALSE | FALSE | TRUE | TRUE |
| T003G12 | EE744297 | iHQNC | FALSE | FALSE | TRUE | TRUE |
| T001G12 | EE744841 | iHQNC | FALSE | FALSE | TRUE | TRUE |
| TImA521 | EE744032 | iHQNC | FALSE | FALSE | TRUE | TRUE |
| T001F08 | EE744675 | iHQNC | FALSE | FALSE | TRUE | TRUE |
| TImA140 | EE745091 | iHQNC | FALSE | FALSE | TRUE | TRUE |
| TImA246 | EE745153 | iHQNC | FALSE | FALSE | TRUE | TRUE |
| TImA391 | EE743920 | iHQNC | FALSE | FALSE | TRUE | TRUE |
| T001E01 | EE744530 | iHQNC | FALSE | FALSE | TRUE | TRUE |
| TImA785 | EE744230 | iHQNC | FALSE | FALSE | TRUE | TRUE |
| T001E09 | EE744575 | iHQNC | FALSE | FALSE | TRUE | TRUE |
| T005C05 | EE744399 | iHQNC | FALSE | FALSE | TRUE | TRUE |
| T001H09 | EE744908 | iHQNC | FALSE | FALSE | TRUE | TRUE |
| T011H01 | EE744901 | iHQNC | FALSE | FALSE | TRUE | TRUE |
| T013H04 | EE745058 | iHQNC | FALSE | FALSE | TRUE | TRUE |
| TImA208 | EE745133 | iHQNC | FALSE | FALSE | TRUE | TRUE |
| T005E04 | EE744421 | iHQNC | FALSE | FALSE | TRUE | TRUE |
| T003H09 | EE744304 | iHQNC | FALSE | FALSE | TRUE | TRUE |
| T010F11 | EE744849 | iHQNC | FALSE | FALSE | TRUE | TRUE |
| T013A07 | EE744984 | iHQNC | TRUE | TRUE | TRUE | TRUE |
| TImA419 | EE743946 | iHQNC | TRUE | TRUE | TRUE | TRUE |
| T001D12 | EE744519 | iHQNC | TRUE | TRUE | TRUE | TRUE |
| Contig122 | a | iHQNC | TRUE | TRUE | TRUE | TRUE |
| T006D01 | EE744494 | Inconsistent | TRUE | TRUE | TRUE | FALSE |
| TImA235 | EE745144 | Inconsistent | TRUE | TRUE | TRUE | FALSE |
| T009F03 | EE744754 | Inconsistent | TRUE | TRUE | TRUE | FALSE |
| TImA745 | EE744198 | Inconsistent | TRUE | TRUE | TRUE | FALSE |
| TImA615 | EE744105 | Inconsistent | TRUE | TRUE | TRUE | FALSE |
| T010H02 | EE744861 | Inconsistent | TRUE | FALSE | TRUE | TRUE |
| TImA482 | EE743998 | Inconsistent | TRUE | FALSE | TRUE | TRUE |
| Contig116 | a | Inconsistent | TRUE | FALSE | TRUE | TRUE |
| T007A07 | EE744547 | Inconsistent | TRUE | FALSE | TRUE | TRUE |
| TImA602 | EE744098 | Inconsistent | TRUE | FALSE | TRUE | TRUE |
| TImA76 | EE744211 | Inconsistent | TRUE | FALSE | TRUE | TRUE |
| Contig8 | a | Inconsistent | TRUE | FALSE | TRUE | TRUE |
| TImA700 | EE744157 | Inconsistent | TRUE | FALSE | TRUE | TRUE |

a

**Contig141**: TImA763 (EE744214), T013A06 (EE744983)

**Contig10**: T003C07 (EE744251), TImA607 (EE744102), TImA404 (EE743934), TImA610 (EE744103), T006B05 (EE744474)

**Contig122**: TImA529 (EE744040), TImA757 (EE744208), TImA389 (EE743919), TImA458 (EE743981), TImA536 (EE744048), T002B07 (EE745041), T013B08 (EE744996), T005C08 (EE744401), T006G01 (EE744526), T009H04 (EE744779)

**Contig116**: TImA465 (EE743986), TImA695 (EE744152)

**Contig8**: T002F03 (EE745163), T008H01 (EE744691)
